# New Amphiphilic Terpolymers of N-Vinylpyrrolidone with Acrylic Acid and Triethylene Glycol Dimethacrylate as Promising Drug Delivery: Design, Synthesis and Biological Properties In Vitro

**DOI:** 10.3390/ijms25158422

**Published:** 2024-08-01

**Authors:** Svetlana V. Kurmaz, Roman I. Komendant, Evgenia O. Perepelitsina, Vladimir A. Kurmaz, Igor I. Khodos, Nina S. Emelyanova, Natalia V. Filatova, Vera I. Amozova, Anastasia A. Balakina, Alexey A. Terentyev

**Affiliations:** 1 Federal Research Center of Problems of Chemical Physics and Medicinal Chemistry, Russian Academy of Sciences, 142432 Chernogolovka, Russia; komendant@icp.ac.ru (R.I.K.); jane@icp.ac.ru (E.O.P.); kurmaz@icp.ac.ru (V.A.K.); n_emel@mail.ru (N.S.E.); natasha55555@yandex.ru (N.V.F.); amozovavi@gmail.com (V.I.A.); stasya.balakina@gmail.com (A.A.B.); alexei@icp.ac.ru (A.A.T.); 2 Institute of Microelectronics Technology and High-Purity Materials, Russian Academy of Sciences, 142432 Chernogolovka, Russia; khodos.igor@mail.ru

**Keywords:** N-vinylpyrrolidone, acrylic acid, triethylene glycol dimethacrylate, radical terpolymerization, amphiphilic terpolymers, functional compound, methylphemophide a, biological activity, cell accumulation, medicinal chemistry

## Abstract

The terpolymers of N-vinylpyrrolidone (VP) with acrylic acid and triethylene glycol methacrylate were synthesized with more than 90% yield by radical copolymerization in ethanol from monomeric mixtures of different molar composition (98:2:2, 95:5: 2 and 98:2:5) and their monomer composition, absolute molecular masses and hydrodynamic radii in aqueous media were determined. Using the MTT test, these terpolymers were established to be low toxic for non-tumor Vero cells and HeLa tumor cells. Polymer compositions of hydrophobic dye methyl pheophorbide a (MPP) based on studied terpolymers and linear polyvinylpyrrolidone (PVP) were obtained and characterized in water solution. Quantum-chemical modeling of the MPP-copolymer structures was conducted, and the possibility of hydrogen bond formation between terpolymer units and the MPP molecule was shown. Using fluorescence microscopy, the accumulation and distribution of polymer particles in non-tumor (FetMSC) and tumor (HeLa) cells was studied, and an increase in the accumulation of MPP with both types of particles was found.

## 1. Introduction

In recent decades, there has been significant research interest in polymer nanoparticles (NPs) based on water-soluble, amphiphilic and insoluble polymers as carriers and means of drug delivery [1,2,3,4,5]. Toxicity, irritation, allergenicity, biodegradability are the main factors determining the possibility of their use in biology and medicine. NPs increase the aqueous solubility of insoluble or poorly soluble biologically active compounds (BAC), ensure their prolonged and controlled release under the influence of external or internal factors, and targeted delivery to the source of diseases. Their small size contributes to the accumulation of drugs in the affected tissue through the mechanism of passive targeting due to the “enhanced permeability and retention (EPR) effect”, which consists of the accumulation of nanoparticles in a tumor having large defects in capillaries, and their slow removal due to the absence of a pronounced lymph flow in it [6,7]. Large molecules or nanoparticles with molecular weight more than 40 kDa accumulate in tumor tissue in a greater amount than in normal ones.

To create nanoparticles, polymers of both natural (starch, alginates, gelatin, chitosan, etc.) and synthetic (polylactide-co-glycolides, polyanhydrides, polycyanoacrylates and polyphthalates, etc.) origin are widely used. The advantages of natural polymers are low cost, biocompatibility, good water solubility, and widespread distribution. Limitations in their use are related to the presence of impurities, variability in polymer composition, and low hydrophobicity to encapsulate lipophilic BAC. Most NPs are produced on the basis of synthetic and semisynthetic polymers, which have a number of advantages: reproducibility of synthesis, stability, the ability to regulate their molecular weight and composition, as well as molecular structure and nature of monomers. Wide opportunities for the design of synthetic macromolecules make it possible to construct NPs that respond to changes in external and internal factors (temperature, pH of the environment, light irradiation, ultrasound, etc.), which allows for the controlled release of BAC into cells and tissues. However, some types of BAC are soluble only in organic solvents and are highly lipophilic; as a result, surfactants should be used to form their stable dispersions. Design and synthesis of polymers as drug-loading cargos assumes their biocompatibility, lack of irritation and allergenicity, biodegradability and water solubility [4]. Amongst the biodegradable polymers, polyglycolic and polylactic acids, their copolymers of various compositions and molecular weights [8,9,10], poly(ε-caprolactone) [4], which hydrolyzes more slowly than polyacids and is suitable for constructing long-acting delivery systems, and polyacrylacetates [11], from which the family of NPs is formed as nanospheres, nanocapsules, etc.

Currently, amphiphilic polymers of complex architecture—dendrimers [12], hyperbranched, star-shaped, etc. [13,14,15,16]—are actively being studied as promising carriers and means of delivering BAC. Dendrimers are considered as ideal delivery systems [17] due to their three-dimensional globular structure, nanosize, monodispersity, lipophilicity, and ability to easily penetrate cells. However, their synthesis requires a complex and multi-step procedure; some of them, for example, containing primary amino groups and a positively charged surface, are highly toxic to cells [18].

Amphiphilic polymers with branched structures may represent an alternative to dendrimers; despite the irregularity and imperfection of architecture, they share similar properties. Due to the topology of polymer chains, they provide prolonged action of drugs, change their distribution in the body, diminish toxicity, etc. [13]. Similar to amphiphilic linear block copolymers, they are self-organized into various supramolecular structures in solutions or at phase boundaries [16,19]. They usually form monomolecular micelles smaller than 10 nm in solution and multimolecular micelles of nano- and micron size. Environmentally sensitive polymer micelles can change volume, structure, and properties under the influence of various factors such as pH, temperature and ionic strength of a solution. Thus, micelles containing thermosensitive chain segments undergo a thermally induced phase transition from a water-soluble to a water-insoluble state, which makes it possible to implement “smart” delivery of BAC. Intracellular or external factors such as changes in pH and temperature are used to control the release of drugs loaded into micelles.

Previously, we synthesized copolymers of N-vinylpyrrolidone (VP) with triethylene glycol dimethacrylate (TEGDM) by radical copolymerization and demonstrated their possibility as biocompatible carriers and delivery vehicles for biologically active compounds of various natures [20,21,22,23]. They are characterized by pronounced amphiphilicity that can be controlled by comonomers nature, their content in macromolecules, sufficiently small size of individual macromolecules and their aggregates in water. It will facilitate their efficient cellular and tissue uptake.

An important factor determining their biomedical use is the ability to respond to external influences, in particular, temperature and pH of the environment [24]. Branched VP copolymers modified with methacrylic acid [23] just belong to such copolymers. In this regard, VP copolymers containing acrylic acid (AA) units capable of ionization are also of scientific and practical interest. So, for example, hydrophilic nanoparticles with polyacrylic acid exhibit pH-dependent dissolution behavior and are favorable for some drugs [25].

To study the penetration of polymeric nanoparticles into cells, we used a commercial zinc tetraphenylporphirinate [21,26] and developed its water-soluble structures based on VP-TEGDM copolymers of 50–100 nm size with intensive fluorescence ranging from 600–700 nm, which had low toxicity and were able to penetrate into Vero and HeLa cells. In addition, a series of nanoparticles with a hydrodynamic radius from 20 to 100 nm was developed over solubilization of hydrophobic dye methyl pheophorbide a (MPP) (chlorin e6 derivative) by some amphiphilic copolymers of N-vinylpyrrolidone with (di)methacrylates and their photophysical properties and biological activity of the NPs aqueous solution were studied as photosensitizers for application in photodynamic therapy (PDT) [27]. Pheophorbide a was found to be the most effective photosensitizer and demonstrates strong absorption at the excitation wavelength as compared with the hematoporphyrin derivative and greater singlet oxygen quantum yield relative to the phthalocyanines and naphthalocyanines [28]. It generates PDT effects both in its monomeric form [28,29] or coupled to DAB-dendrimers and fullerenes [30,31] and pheophorbide a/HAS (human serum albumin) nanoparticles [32,33].

In complex systems of the “guest-host” type, the nature of the interaction between the components depends on their chemical composition, local structure and solvent. Encapsulation of a guest molecule inside the cavity of a macromolecular system is due to dispersion, electrostatic interactions and hydrogen bonds. Prediction of encapsulation energies, final structures and elucidation of the nature of intermolecular forces between the host and the guest molecules is possible using quantum chemical modeling methods. They can be very useful to establish the electronic structure of active compounds and intermolecular interactions in their systems with a polymer, see, e.g., [34,35,36]. The latter is very important for predicting the possibility of the existence of such structures and their application in vivo. Thus, in the work [12] by approaches based on DFT, the possibilities to use dendritic macromolecular structures as carriers of doxorubicin were investigated, and the encapsulation energy of the complexes “host-guest” suggests that these systems could be used in pharmacological applications.

The goals of this work are to synthesize new amphiphilic terpolymers of N-vinylpyrrolidone with acrylic acid and triethylene glycol dimethacrylate by conventional radical polymerization as one-pot simple method, to determine their main physicochemical characteristics (monomer composition, absolute weight-average molecular weight with polydispersity and hydrodynamic radius in water solutions), and also estimate their cytotoxicity and ability to penetrate and accumulate non-tumor Vero/FetMSC and tumor HeLa cells. An important aspect of this work is the quantum chemical modeling of possible structures formed as a result of intermolecular interactions of MPPs and terpolymer units. Further important aspects of this study include an assessment of the possibility to form hydrogen bonds between electron donors of copolymer units and proton donors of the MPP molecule, and their quantitative characterization.

## 2. Results and Discussion

### 2.1. Synthesis of VP-AA-TEGDM Copolymers, Their Structure, Physicochemical Characteristics and Cytotoxicity In Vitro

VP-AA-TEGDM terpolymers were synthesized in ethanol from monomer mixtures with molar compositions of 98:2:2 (CPL1), 95:5:2 (CPL2), and 98:2:5 (CPL3). Linear polyvinylpyrrolidone (PVP) was obtained as well. The microstructure of the studied terpolymers can be assessed by literature and our own data on the reactivity of comonomers. Ordinary constants of binary copolymerization are relative activities of monomers in terpolymerization. In this regard, based on the literature data on the binary copolymerization of VP, MMA as a linear analogue of TEGDM and AA, conclusions can be drawn about the distribution of VP, AA and TEGDM units in VP-AA-TEGDM terpolymers. We have shown [20] that TEGDM is more active in radical copolymerization than VP. This fact is consistent with data on the relative activities of MMA and VP [37,38]. In turn, according to [39], the binary copolymerization constants *r*_1_ of MMA (M_1_) and *r*_2_ AA (M_2_) are 1.25 and 0.225, respectively, i.e., methacrylate is more active than AA. Since *r*_1_ × *r*_2_ < 1, the distribution of these units in the MMA-AA copolymer is statistical. Other methacrylates also show higher activity. For example, the relative activities of AA and methoxypolyethylene glycol methacrylate in water were 0.02–0.07 and 1.14–1.20, respectively [40]. Thus, provided that TEGDM and MMA are equally active, the bifunctional monomer is also more reactive than AA. In turn, VP (M_1_) is more reactive than AA (M_2_) in binary copolymerization, and taking into account the copolymerization constants *r*_1_ = 2.46 and *r*_2_ = 0.179, a statistical VP-AA copolymer is formed [41]. Based on this data, it can be assumed that the polymer chain at the initial stages consists of statistically distributed units of VP, TEGDM and AA. At deep stages, PVP chains formed from the monomer, which is in excess, are attached to the “pendant” C=C bonds of TEGDM units, i.e., VP or chains consisting of VP and AA units. The result is a 3D structure with branches in the polymer chains; both VP and AA units may be present at the ends of polymer chains.

**Figure 1:**
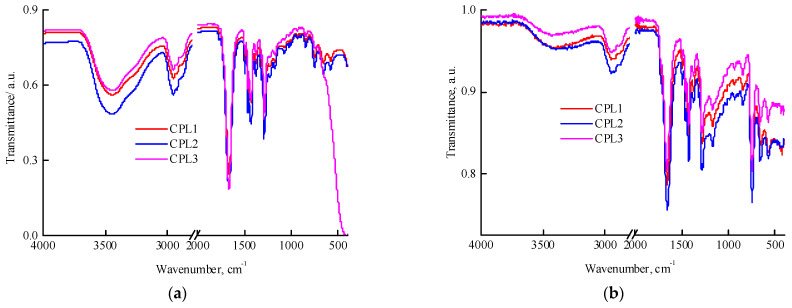
IR spectra: films of CPL1-CPL3 cast from chloroform (**a**), and powders of CPL1-CPL3 fractions insoluble in chloroform (**b**).

All terpolymers are soluble in polar media like DMSO, N-methylpyrrolidone, ethanol and water but poorly soluble in low-polar media such as THF and chloroform. The IR spectra of films of a VP-AA-TEGDM terpolymer fractions partially dissolved in chloroform (Figure 1a) and powders insoluble in this solvent (Figure 1b) were recorded. In films cast from chloroform, an intense absorption band was observed, attributed to the C=O stretching vibrations of VP groups with a wavenumber of ~1660 cm^−1^. In the spectrum of the CPL3, the C=O group of TEGDM units is presented as a shoulder at 1719 cm^−1^ and is poorly resolved. It is not possible to identify AA units in this region of the spectrum due to overlapping with the TEGDM and VP absorption band corresponding to C=O vibrations; the characteristic stretching vibrations of OH groups within the region of 3000–3600 cm^−1^ are overlapped by the absorption of adsorbed water hydrogen bonded to the copolymers [42]. However, absorption in 3200–3000 cm^−1^ region indicates the presence of COOH groups in studied terpolymers. Figure 1b shows the IR spectra of the fractions insoluble in chloroform that are distinguished with higher content of (di)methacrylates, as evidenced by increasing in the absorption region of the stretching vibrations of methacrylic C=O groups and contain significantly less water, i.e., differ in composition and degree of hydrophilicity from the fraction soluble in chloroform.

**Figure 2:**
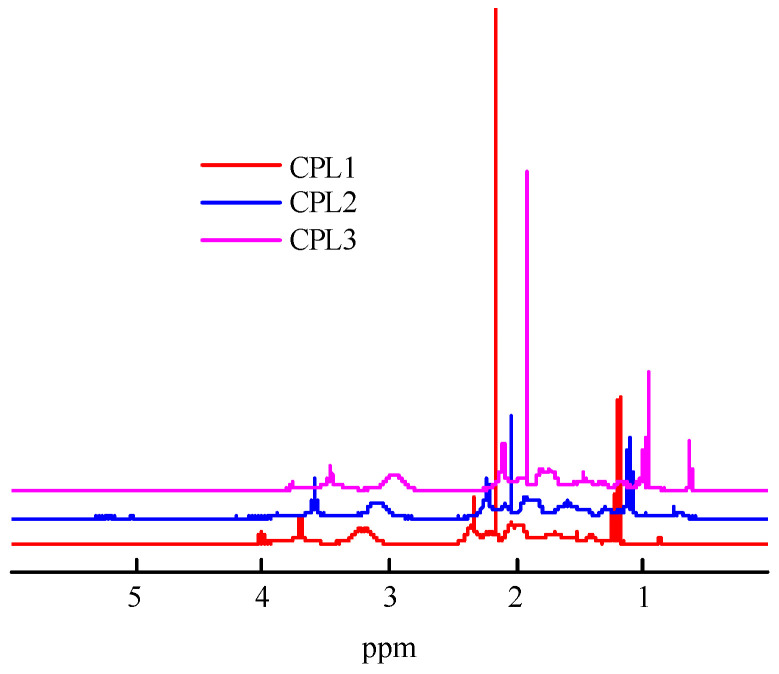
^1^H NMR spectra of CPL1-CPL3 in deuterated chloroform. The terpolymer concentrations were 8.3, 7.5, and 7.9 mg mL^−1^, respectively. The spectra are normalized to the solvent signal. The ppm values were shifted on 0.125 and 0.25 for CPL2 и CPL3, respectively.

Figure 2 shows the ^1^H NMR spectra of the CPL1-CPL3. The main signals were described as the VP units [43], constituting a major portion of terpolymers. The spectra show two groups of signals corresponding to VP units. The first group includes signals at δ3.0–4.0 ppm due to NCHα protons of polymer chains and CH_2_C=O moieties of pyrrolidone. The second group is composed of signals at δ1.4−2.4 ppm related to protons of CH_2_ moieties in polymer chains and C–CH_2_–C and NCH_2_ moieties of pyrrolidone. The signals attributed to C=CH_2_ units were not observed, showing a high conversion for these groups. The proton present in the carboxylic group of AA unites resonates at δ11.2 ppm [41]. But they do not appear in the ^1^H NMR spectra of all terpolymers due to the low concentration of AA units. The signal at ~δ2.4 was attributed to the protons of water molecules.

Using elemental analysis, the N content in the resulting VP-terpolymers was determined (Table 1). The terpolymers contain nitrogen, a characteristic of VP units. Based on N content data, the monomer compositions of terpolymers were calculated.

Figure S1 shows chromatograms of CPL1-CPL3 obtained from two detectors—a refractometer and light scattering data. It can be seen that CPL3 contains a high-molecular-weight fraction, in contrast to the other terpolymers, which indicates the presence of a highly branched fraction in this terpolymer. Molecular mass characteristics—absolute weight-average molecular masses M_w_ and polydispersity PD are given in Table 1. From these data, it follows that an increase in the AA content in the monomer mixture leads to a ~2-fold decrease in the molecular weight of the CPL2. An increase in the TEGDM content in the monomer mixture under CPL3 preparation leads to a sharp increase in the M_w_ and PD values of the resulting terpolymer apparently due to its high degree of branching. The highly branched copolymers synthesized by radical copolymerization have a wide molecular mass distribution due to their statistical nature [44]. To control the molecular mass distribution of VP-TEGDM copolymers, we used 1-decanethiol as regulator of polymer chain length [20]. However, its residues are included in polymer chains and pollute the copolymer by sulfur-containing compound. As a consequence, copolymers’ production is complicated and their cost rises; moreover, their special purification is necessary.

**Figure 3:**
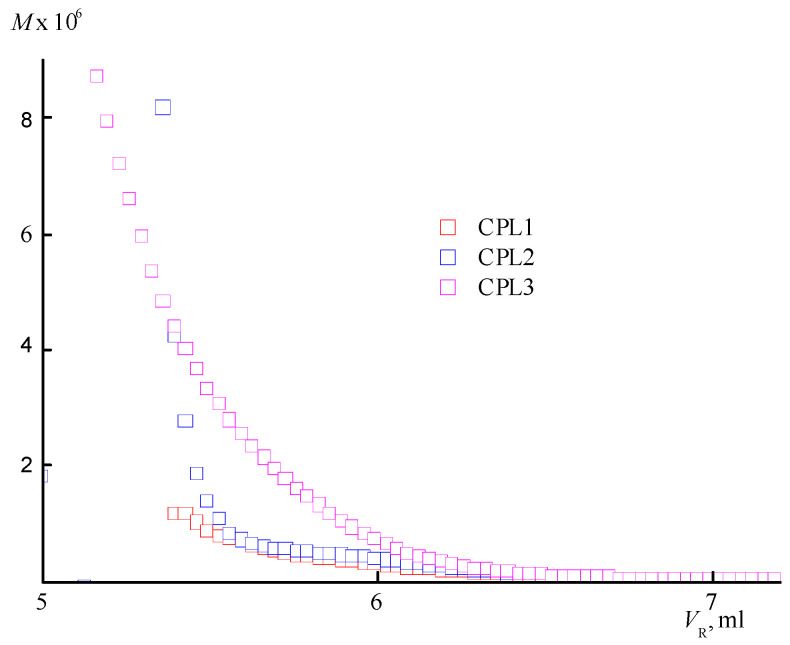
The molecular weight dependencies of CPL1-CPL3 on the eluent volume *V_R_*.

An increase in the degree of branching with increasing TEGDM content of the CPL3 is evidenced by an analysis of the dependencies of the molecular weight (*M*) on the eluent volume *V_R_* (Figure 3). It is located above similar dependence for the copolymers CPL1 and CPL2. At the same *V_R_* value, CPL3 macromolecules with a higher molecular weight and degree of branching are eluted. Based on the SEC data, it can be assumed that the CPL1 and CPL2 are weakly branched or have the structure of nanogels like cross-linked cyclic structures. AA units promote aggregation of polymer chains due to a strong intermolecular interactions, which favors intra- and intermolecular cross-linking reactions.

**Figure 4:**
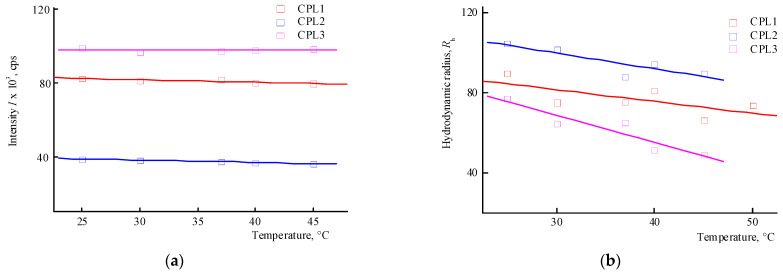
Dependencies in the average intensity of light scattering (**a**) by aqueous buffer saline solutions (pH 7.4) of CPL1-CPL3, and the average hydrodynamic radius R_h_ of their dispersing centers (**b**) from temperature. The terpolymer concentrations were 5 mg mL^−1^.

The behavior of CPL1-CPL3 in water and PBS was investigated by dynamic light scattering (DLS) method in the range of 20–50 °C. The distribution of the intensity of light scattering by the size of the scattering centers by aqueous buffer solutions (pH 7.4) of terpolymers is bimodal. The main contribution in it is made by scattering centers, the R_h_ values of which at 25 °C lies in the interval of ~80–100 nm (Table 1). Figure 4a shows the dependencies of the average intensity of light scattering by CPL1-CPL3 aqueous PBS on temperature. It can be seen that in the studied temperature interval, the intensity of light scattering practically does not change, i.e., their clouding point lies above 50 °C. In this case, the size of the main type of dispersing centers decreases (Figure 4b). It is known [39] that the pK_a_ of AA is 4.25 in water and its units in the terpolymers will be in partially ionized form. Accordingly, the proportion of this form will increase in PBS.

**Figure 5:**
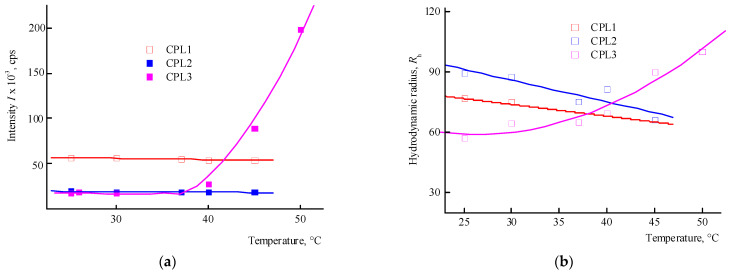
The dependencies of the average intensity of light scattering (**a**) by aqueous solutions (pH 6) of CPL1-CPL3 and the average hydrodynamic radius R_h_ of their dispersing centers (**b**) from temperature. Terpolymer concentration was 2 mg mL^−1^.

The distribution of the intensity of light scattering by aqueous solutions (pH 6) on scattering centers of CPL1-CPL3 is bimodal as well. Figure 5 shows the dependencies of the average intensity of light scattering by aqueous solutions of CPL1-CPL3 and the average hydrodynamic radius R_h_ of their dispersing centers on temperature. It can be seen that the aqueous solutions of CPL1 and CPL2 do not respond the temperature change, like in buffer solutions. Perhaps this is due to the topological structure of their nanogel particles and strong intramolecular interactions of polymer chains. However, with an increase in temperature, the intensity of the light scattering of the aqueous CPL3 solution increases, i.e., this terpolymer is thermo-sensitive. The growth in intensity occurs at ~40 °C, and the aqueous solution begins to become cloudy. This process is reversible—when cooling to room temperature, the solution again becomes transparent. In work [42], we showed that water molecules bond with the oxygen C=O of VP lactam ring, ether and carbonyl groups of TEGDM units. The addition enthalpy (−∆*H*) of one water molecule on the C=O groups of VP units for the VP-VP-VP copolymer moiety is 4–6 and 2–4 kcal mol^−1^ on the ether and carbonyl groups of the TEGDM unit [42]. The H-bonds between (di)methacrylates units and water are primarily destroyed with increasing temperature, and the solubility of the copolymer decreases.

Thus, among the polymers studied, only CPL3 with a high molecular weight and content of TEGDM units is thermo-responsive in water. However, its cloudy point in PBS is shifted to higher temperature values. The lack of a terpolymer aggregation is due to the presence of charged carboxyl groups of the AA units, which apparently leads to the expanding of polymer chains and stabilizing their solutions.

**Figure 6:**
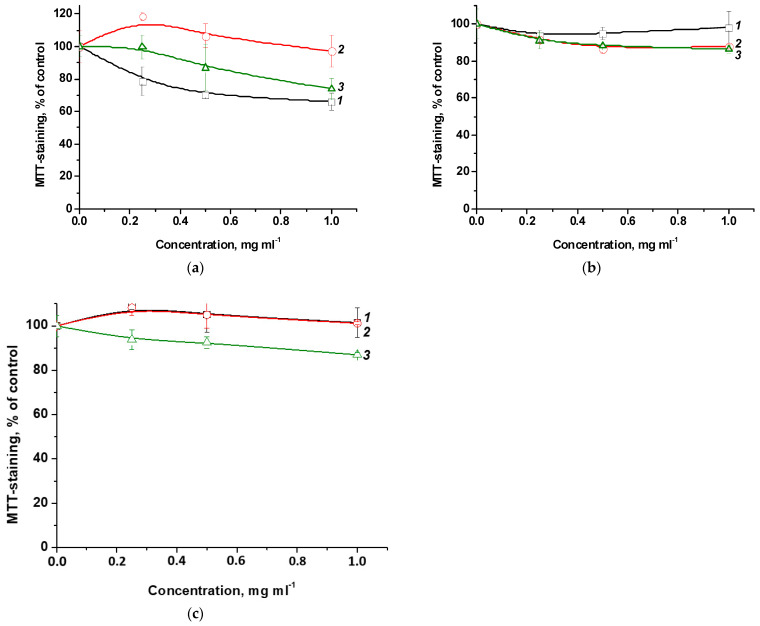
Effect of terpolymers CPL1 (curves *1*), CPL2 (curves *2*) and CPL3 (curves *3*) with exposure for 72 h on the viability of FetMSC (**a**), Vero (**b**) and HeLa (**c**) cells according to the results of MTT staining.

The studied terpolymers may be promising in various biomedical applications, in particular, as carriers and drug delivery vehicles. In this regard, their cytotoxic properties were studied using a model of mammalian cells in vitro. Figure 6 shows the dependence of MTT staining of cells of various origins on the concentration of terpolymers with as long exposure as 72 h. One can see that in the studied concentration range, terpolymers exhibit low cytotoxicity or do not affect cell viability at all. So, the viability of the most susceptible FetMSC cells at the maximum concentration of CPL1 terpolymer (1 mg mL^−1^) decreases by ~30% (Figure 6a). For Vero and HeLa cells, a decrease in viability under the influence of CPL3 by ~10% was observed (Figure 6b,c). Thus, terpolymers in which the main component is the biocompatible VP monomer are low in toxicity in the studied concentration range and can be used in biomedical applications as an inert platform for drug delivery.

### 2.2. Polymer Compositions of Methyl Pheophorbide as a Hydrophobic Dye and Their Characterization

The process of encapsulation of MPP into terpolymer NPs is based on the mechanism of their intermolecular association. MPP limited solubility in isopropyl alcohol (IPA) promotes association with NPs, which occurs due to the physical capture of dye molecules by the polymer matrix with penetration into the internal cavities of NPs. As a consequence, “guest-host” complexes are formed, in which hydrophobic interactions between dye molecules and low-polar regions of polymer chains stimulate the penetration of “guest” molecules in these moieties of individual macromolecules and their aggregates.

CPL1-CPL3 terpolymers consist of hydrophilic VP and AA units and hydrophobic TEGDM units; their macromolecules can be considered as structures in which there are regions that differ significantly in polarity and are formed by moieties of polymer chains consisting of TEGDM, VP and AA units. To achieve thermodynamic stability by reducing the hydrophobic interaction of non-polar groups and water molecules, individual amphiphilic macromolecules adopt a like core-shell form, where hydrophobic regions assemble into regions protected by a hydrophilic shell. Hydrophobic compounds can be easily encapsulated into such structures. Here we used the water-insoluble dye MPP, which exhibits high fluorescence in the red region of the spectrum, to obtain its water-soluble structures and study their penetration and accumulation in various cells.

The dye was encapsulated Into terpolymers using a simple and efficient method [26,27]. At the first stage, solutions of the terpolymers in isopropyl alcohol were used to maintain the homogeneity of the medium and prevent self-aggregation of macromolecules and large particles formation. According to DLS, terpolymers exist in an alcohol solution mainly as individual macromolecules with R_h_~4 nm. The dye was dissolved in toluene or DMSO. After introducing a solution of the dye into the terpolymer solution and removing the solvents, polymer compositions of MPP were obtained, which easily dissolved in water or PBS to form transparent or opalescent systems, depending on the type of terpolymers and PC concentration.

Figure S2 shows absorption spectra of MPP solutions of different concentrations in toluene and DMSO. There is an intense absorption Q-band in the visible region with a maximum at wavelengths of 675 and 672 nm *S*_0.0_→*S*_1.0_ transition [33] in toluene and DMSO, respectively. The dependencies of its optical density on concentration in the range less than 10^−4^–10^−5^ M in both the solvents are linear, and the molar extinction coefficient is equal to 54,438 and 38,898 L/M × cm, respectively. However, MPP molecules aggregate over time, and the A([MPP]) dependence becomes nonlinear; the maximum of the absorption band shifts to 668 nm as a result of MPP aggregation in the polar medium.

#### 2.2.1. Polymer Compositions of MPP (PC1-PC4)

**Figure 7:**
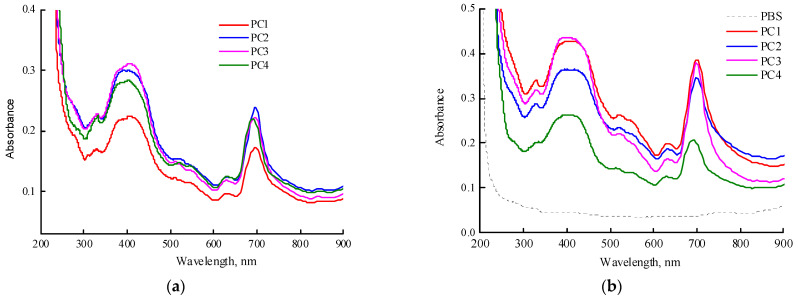
Absorption spectra of freshly prepared solutions of PC1-PC4 in water (**a**) and in PBS (**b**). Concentration of MPP compositions in water is ~1 mg mL^−1^. Cuvette was 1 cm.

Encapsulation of MPP into polymer particles CPL1-CPL3 was carried out to prepare PC1-PC3 compositions. For comparison, a composition based on PVP (PC4) was prepared as well. Figure 7a shows the absorption spectra of freshly prepared aqueous solutions of MPP terpolymer compositions (~1 mg mL^−1^). It should be noted that PC1, PC2, and PC4 dissolved quickly, in contrast to PC3. The maximum of the Q-absorption band of encapsulated MPP in water was 698 nm and, compared to one in toluene and DMSO, was red-shifted by more than 20 nm. It should be noted that the maximum absorption band of MPP encapsulated in PVP was about 690 nm, i.e., differed by 8 nm from the MPP encapsulated in terpolymers.

However, the absorption spectra of the solutions of all studied systems changed over time (Figure S3). Thus, in an aqueous solution of PC1, the absorption band continued to shift to the red region of the spectrum and its optical density decreased. In an aqueous solution of PC2 in the visible region, the entire absorption spectrum decreased as a result of phase precipitation from the colloidal solution, but the position of the maximum of the absorption band remained the same. In the case of PC2, an increase in the optical density of the absorption band was observed, apparently as a result of an increase in the solubility of the original MPP. In the spectrum of the aqueous solution of PC4, the changes were the most significant. The maximum of the absorption band first shifted to 698 nm, its optical density decreased and the optical density of the MPP absorption bands at 634 and 757 nm increased, probably as a result of the appearance of different aggregates (H- and J-aggregates) either inside or on the surface of nanoparticles. Thus, in aqueous solutions of nanostructures, significant changes occur over time, associated with a slight precipitation of large NPs-MPP, an increase in the interaction of MPP-polymer, and also aggregation of encapsulated MPP molecules in NPs. The PC4 structures were less stable than those based on terpolymers; H- and J-aggregates increased over time. Vice versa, PC3 structures based on CPL3 with a high molecular weight and content of TEGDM units are the most stable in water; in them, the aggregation of MPP molecules is suppressed, but the interaction with the terpolymer is enhanced and is accompanied by a hyperchromic effect (Figure S3c). The content of H- and J-aggregates increased over time.

Figure 7b shows absorption spectra of freshly prepared solutions of PC1 (0.98 mg mL^−1^), PC2 (1 mg mL^−1^), PC3 (0.86 mg mL^−1^), and PC4 (1.08 mg mL^−1^) in PBS. The same features as described above for water solutions are observed in this medium. The maximum Q-band absorption of MPP encapsulated in CPL1-CL3 is observed at a wavelength of 698 nm, in contrast to PVP itself, for which it is 690 nm. These differences are probably associated with the localization of MPP molecules in regions of NPs that provide strong interaction between the functional groups of MPP and terpolymers. However, over time, these trends also increase in PVP-based NPs.

To calculate the MPP content in compositions (the MPP loading), they were dissolved in DMSO and solutions containing 1.0, 1.1, 0.96 and 0.86 mg mL^−1^ PC1-PC4 were obtained, respectively. The absorption spectra of the freshly prepared solutions were recorded and the absorption band of MPP in DMSO was observed at 672 nm, similar to that in DMSO (Figure S4) as a result of its release from polymer nanoparticles. Knowing the molar extinction coefficient of MPP in DMSO and the concentrations of MPP composition in DMSO, the MPP loading was calculated. According to calculations, they were 1.76, 1.55, 1.61 and 1.28%, in PC1-PC4, respectively. The theoretical MPP loading were 1.53, 1.46, 1.47 and 1.35%, respectively. Taking into account experimental errors, it can be assumed that the efficiency of MPP encapsulation in terpolymers is *ca* 100%, and it is about 94.8% in the case of PC4. The efficiency of encapsulation was calculated as the encapsulated mass of MPP by the total mass of the dye in the MPP-composition preparation. It should be noted that after 3 months, significant changes occurred in solutions of MPP composition in DMSO. The solutions remained clear and no phase separation was observed. However, the optical density of the dye absorption band decreased by 60–70% due to MPP release from NPs to DMSO, and aggregation of its molecules. The appearance of absorption in the region of 700 nm indicated the formation of J-aggregates of MPP in this solvent.

The sizes of scattering centers of PC1-PC4 depending on their concentrations in the PBS were determined by the DLS (Table S1). It can be seen that the solutions contain fairly large particles, the size of which decreases with concentration; this indicates the aggregative nature of these formations. Hydrophobic MPP molecules included in NPs enhance their hydrophobicity, which increases their tendency to aggregation. It is possible that some MPP molecules adsorbed on the surface of NPs can form supramolecular clusters, connecting individual nanostructures with each other. In dilute solutions of PC2 and PC3, the size of scattering centers is higher measuring about 190 and 140 nm, respectively, and decreases after solution filtration. The reason for this is the increase in hydrophobicity of the original polymer nanoparticles and MPP structures based on them.

**Figure 8:**
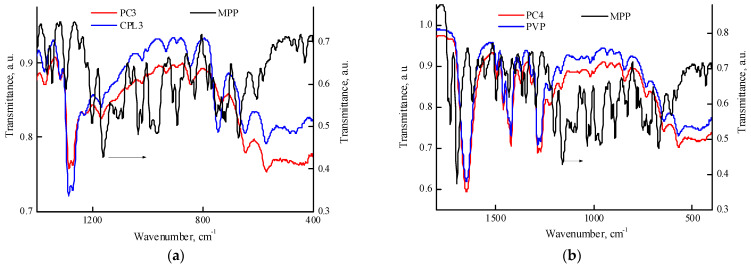
IR spectra of the powders: MPP, CPL3, PC3 (**a**); MPP, PVP and PC4 (**b**).

Figure 8 contains IR spectra of MPP, PVP, PC4, CPL3 and PC3 powders within 1700–400 cm^−1^ region. It can be seen from Figure 8a that the absorption band at 744 cm^−1^ is shifted to lower wavenumbers and its optical density changes in comparison with the initial terpolymer; the absorption band at 571 cm^−1^ has a pronounced shoulder at 556 cm^−1^. Similar shifts of the absorption band are observed in the IR spectra of PC1 and PC2 powders. These changes indicate the presence of interaction between the terpolymer and MPP in the solid phase. Figure 8b shows the IR spectra of PVP, PC4-MPP and MPP powders in the region of 1800–400 cm^−1^. One of the most intense MPP absorption bands is visible at a wavenumber of 1740 cm^−1^. However, a shift in the absorption band in the region of 740 cm^−1^, related to skeletal vibrations, as in the case of terpolymers, is not observed. This allows us to assume that MPP is localized in the sites of terpolymer formed by (di)methacrylate units.

#### 2.2.2. Polymer Compositions of MPP (PC5-PC7)

**Figure 9:**
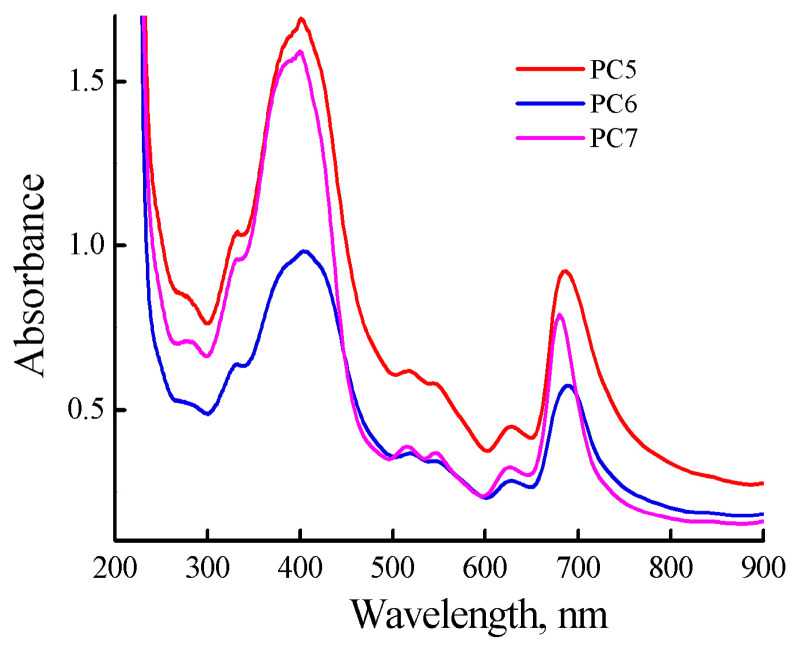
Absorption spectra of freshly prepared solutions of PC5-PC7 in PBS. The PC5-PC7 solution concentrations were 1.1, 0.98, 1.1 mg mL^−1^.

Encapsulation of MPP into polymer particles was carried out according to the method described in the Experimental Section; here, we used a solution of MPP in DMSO (0.8 mg mL^−1^) and calculated MPP content per copolymer was higher than 3%. The resulting MPP compositions based on CPL1, CPL2 and CPL3 (PC5-PC7) were easily dissolved in PBS (1.1, 0.98, 1.1 mg mL^−1^); all the solutions were transparent. The absorption spectra of freshly prepared solutions were recorded (Figure 9). The Q-band absorption is higher than in Figure 7b, due to the higher content of MPP in the obtained compositions. There are differences in the position of the absorption band maximum depending on the type of polymer matrix. Changes occur in all solutions over time. Thus, in solution of PC5, the absorption band of MPP shifts to the red region of the spectrum from 683 to 694 nm and its absorption decreases. In the PC6 solution, the MPP absorption band also shifts to the red region of the spectrum to 698 nm during the same observation time, but its absorption decreases slightly. In the PC7 solution, the position of the maximum of the absorption band does not change, but its absorption decreases and additional absorption appears in the region of 700–750 nm, characteristic of MPP J-aggregates.

To calculate the content of MPP in PC5-PC7 (the MPP loading), their solutions in DMSO were prepared and the absorption spectra were recorded; the calibration dependence of the optical density of the absorption band on the concentration of MPP in DMSO was used as well. According to experimental data, the MPP loading in PC5 was 3.27%, and in PC6 and PC7 were only ca. 2.5 and 2.2%, respectively. The calculated content of MPP in these compositions (MPP loading) was 3.28, 3.15 and 3.10% MPP. The encapsulation efficiency in the first case was ~100%, in the others—80 and 71%, respectively. However, the data on the MPP loading in PC6 and PC7 appear to be of estimation due to possible incomplete release from polymer matrices CPL2 and CPL3 characterized by a high molecular packing density compared to CPL1, as well as intermolecular interactions with terpolymers.

As in aqueous solutions, changes also occur in the spectra of solutions of MPP compositions in DMSO over time. However, they manifest themselves only in a decrease in the optical density of the Q-band at 672 nm by ~30–40% and the appearance of slight absorption in the region of 700–720 nm, associated with the J-aggregates of MPP molecules.

Table S2 shows the sizes of the main scattering centers of PC5-PC7 in PBS at 25 °C. It can be seen that they weakly depend on the PC concentration in the solution. As expected, their size decreases after filtering of dilute solutions. However, their tendency to aggregation is so high that in a solution filtered from large particles, micron-sized particles are still recorded as a result of PC aggregation.

Figure S5 shows the IR spectra of MPP and PC5 and PC7 powders in the regions of 1400–400 cm^−1^. It can be seen that in the IR spectrum of the PC5 powder (Figure S5a), the CPL1 band at 744 cm^−1^ shifts to the region of lower wavenumbers, and the spectrum in the region of 750–400 cm^−1^ changes due to the redistribution of the intensities of the absorption bands in this region. A similar shift occurs in the PC6 composition. Even more pronounced changes are observed in the IR spectrum of PC7 powder (Figure S5b). They are the result of the MPP influence on the vibrational structure of terpolymers, associated not only with a shift in the absorption band but also with the presence of new bands, and are not associated with the residual solvent—DMSO.

### 2.3. TEM Analysis of PC1 and PC3 from Water Solution

**Figure 10:**
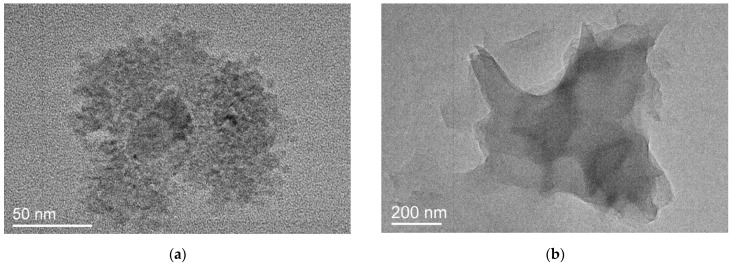
A particle that is a conglomerate of small amorphous particles (**a**) and a “monolithic” amorphous particle (**b**).

In dried PC1 samples, particles of various shapes and structures are observed. Particles can be either amorphous or highly ordered. Figure 10a shows an amorphous particle, which is an agglomerate of round particles about 10 nm in size. Figure 10b shows an image of an amorphous “monolithic” particle.

**Figure 11:**
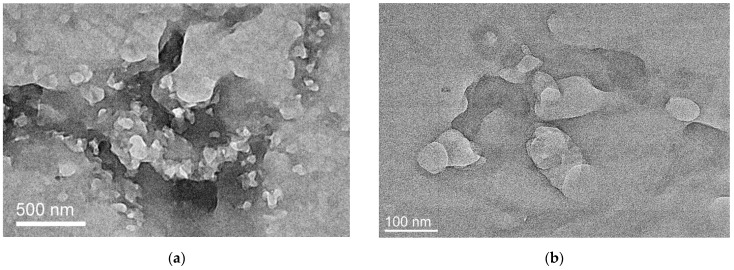
Bright areas in a PC1 sample deposited from water solution at various magnifications (**a**,**b**).

In Figure 11 it can be seen that, along with contrasting formations, there are many amorphous lighter local areas. Perhaps they correspond to cyclic structures of the PC1 copolymer with a low substance content in the volume. Their appearance may also be associated with the destruction of the polymer film on the substrate upon drying since the original terpolymer has a low molecular weight and weak physical and mechanical properties.

**Figure 12:**
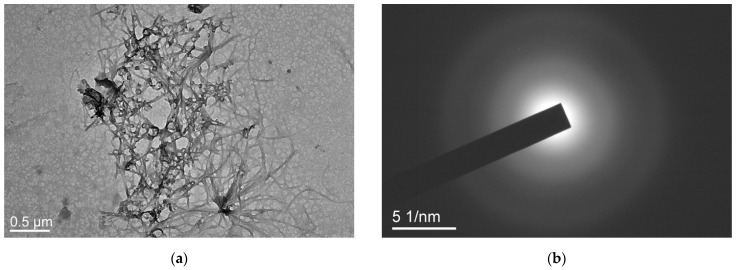
Images (**a**) and diffraction pattern (**b**) of threadlike structures.

Figure 12a shows images of filamentous formation of an amorphous nature, as well as polymer particles associated with them. The diffraction pattern in Figure 12b indicates the amorphous state of these structures. The presence of similar structures is also typical for the PC1 sample and is associated with the peculiarities of the molecular structure and composition of these terpolymers.

**Figure 13:**
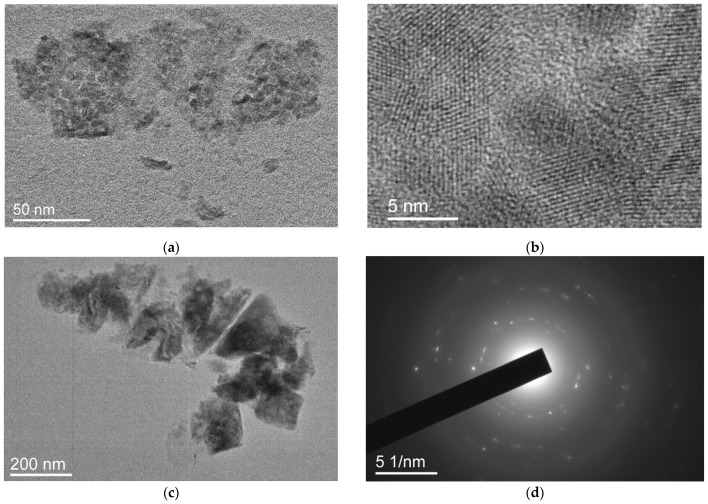
Highly ordered particles in sample PC1: (**a**) a conglomerate of small particles, (**b**) lattice resolution image of such particles, (**c**) large “monolithic” particles and (**d**) the corresponding diffraction pattern.

In the dried PC1 sample, as in the PC3 sample, there are also particles with a highly ordered structure. Figure 13a shows both individual highly ordered nanoparticles 5–10 nm in size and their conglomerates of various sizes. Figure 13b shows the ordered structure of these particles. There are other types of conglomerates with an ordered structure. Figure 13c shows an example of larger particles of various shapes with signs of “crystallinity”, as shown by the diffraction pattern in Figure 13d.

**Figure 14:**
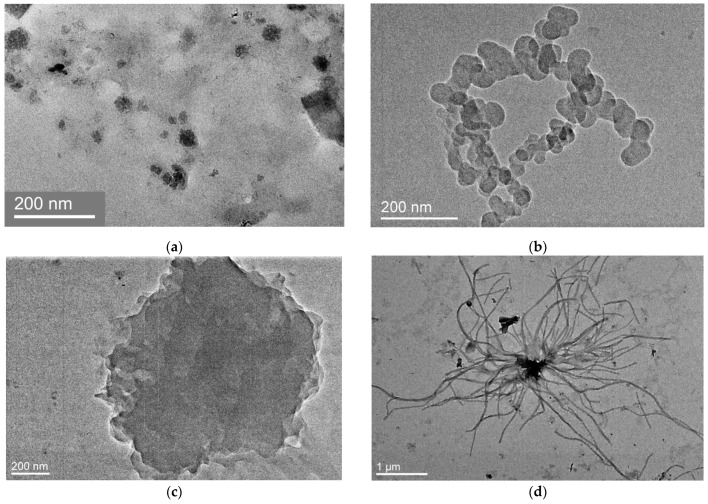
TEM images of amorphous particles in the PC3 sample. (**a**) Single nanoparticles; (**b**) conglomerate of particles smaller than 100 nm; (**c**) single large amorphous particle; (**d**) filamentous formation with a nucleus.

As in the PC1 sample, the PC3 sample precipitated from aqueous solution has a large number of different nanoparticles (Figure 14). They are presented as single particles (Figure 14a) or conglomerates consisting of particles smaller than 100 nm (Figure 14b). Amorphous particles can represent bulk formations and reach significant sizes, such as the particle in Figure 14c. In addition to the characteristic 3D nanoparticles for this range of polymers loaded with bioactive compounds [43], we found unusual micron-sized structures (Figure 14d) with a contrasting core and filamentous formations. In the core of such a structure, MPP molecules are localized, enhancing its contrast. It is possible that this represents the structure of a “super polymeric micelle” with a core of densely packed low-polarity chains and linear chains. A shell is formed by hydrated polar chains of PVP and is characteristic of amphiphilic copolymers of VP [42]. According to calculations, in addition to the first hydrate shell, there is a second shell formed by water molecules bound by hydrogen bonds with each water molecule from the first hydrate shell. Weakly bound water molecules evaporate in the process of sample preparation, while water, firmly bound to the polar groups of the copolymer, forms a shell of polymer particles, which does not collapse when drying due to the long chain length.

**Figure 15:**
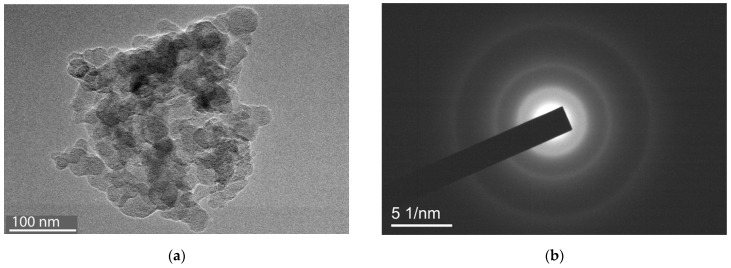
Particle in the PC3 sample consisting of ordered regions of several tens of nanometers in size (**a**) and corresponding diffraction pattern (**b**).

Along with amorphous particles, particles with signs of “crystallinity” were detected (Figure 15). This is evidenced by diffraction rings on the electronogram in Figure 15b. The diffuse (blurred) appearance of these rings shows that the ordered regions are of the order of 10 nm in size and have a chaotic orientation. The orderliness of the particle structure may be related to the conformation of the hydrated PVP chains that form the shell and constitute the basis of the terpolymers. It is known [45] that in hydrated PVP the first water molecule forms two H-bonds with oxygen atoms of two pyrrolidone rings. As a result, the distance between the rings decreases to ~4.2 Å. The convergence of the pyrrolidone rings leads to straightening of the chain sites upon interaction with water. This mechanism of the appearance of highly ordered regions is probably realized in the studied copolymers as well.

### 2.4. Quantum Chemical Modeling of MPP Structures with Terpolymers

**Figure 16:**
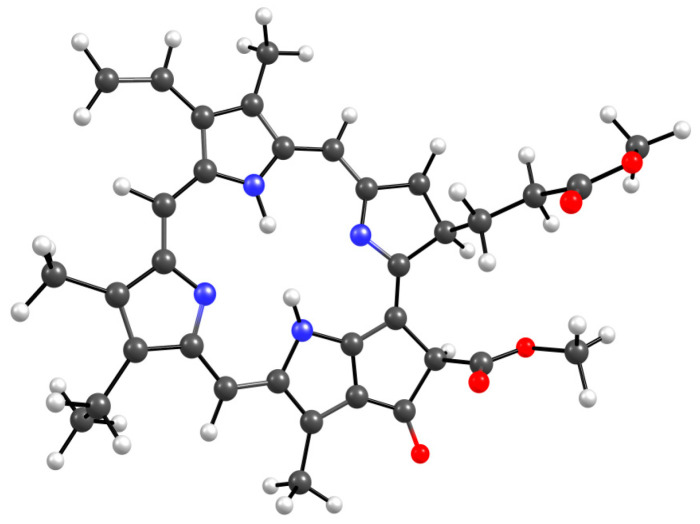
Optimized MPP geometry.

Analysis of the absorption spectra shows that specific processes occur in aqueous solutions of PC, associated not only with the aggregation of NP-MPP and MPP, but also with subtle intermolecular interactions between the terpolymer and the dye molecules. In this regard, we carried out quantum chemical modelling of possible structures formed due to intermolecular interactions between MPP and terpolymers chains. Figure 16 shows the optimized MPP geometry.

Several groups of hydrogen atoms can take part in the formation of hydrogen bonds with copolymer units, all of them have approximately the same partial positive charge, ca. +0.2, except the hydrogen atoms in close proximity to the nitrogen atoms, for which the charge is +0.42, that is, this position would be the most likely for the formation of an intermolecular bond if not for the steric hindrances that may arise. The MPP oxygen atoms can also form bonds with the hydrogens of the VP and TEGDM units, and especially with the protons of the MAA units [46].

**Figure 17:**
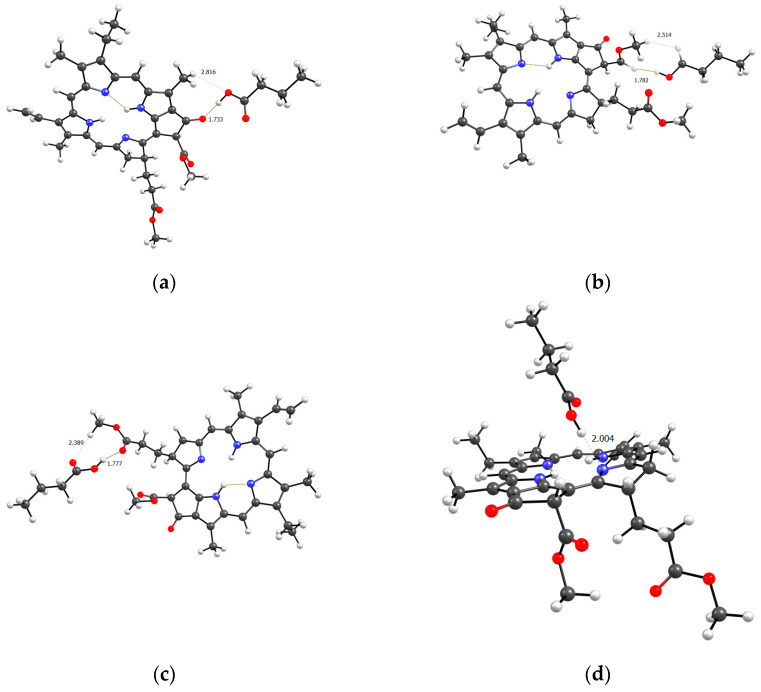
Optimized geometries of MPP dimers with AA (**a**–**d**) units of VP-AA-TEGDM terpolymer.

**Figure 18:**
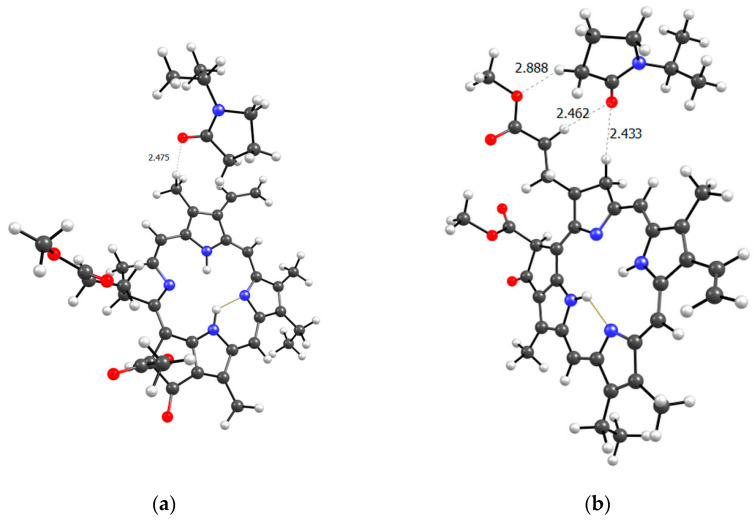

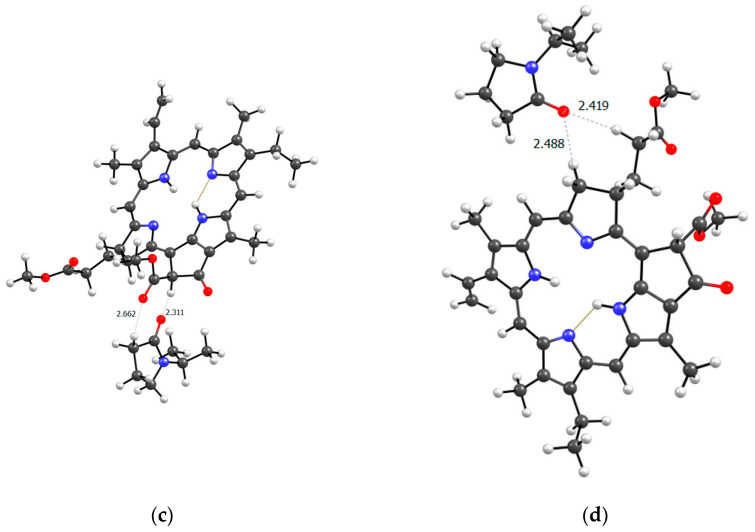
Optimized geometries of MPP dimers with VP (**a**–**d**) units of VP-AA-TEGDM terpolymer.

**Figure 19:**
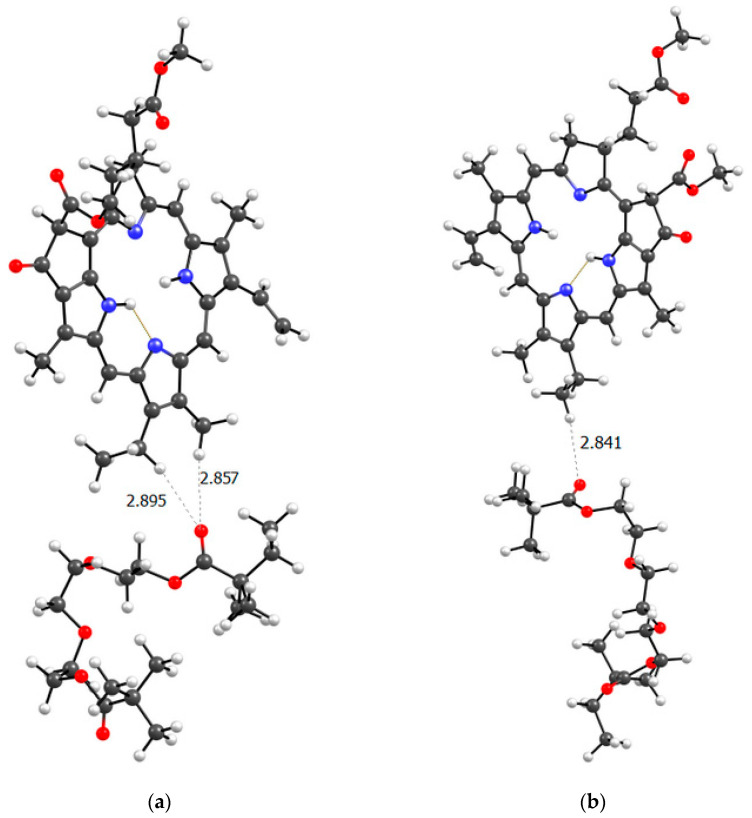

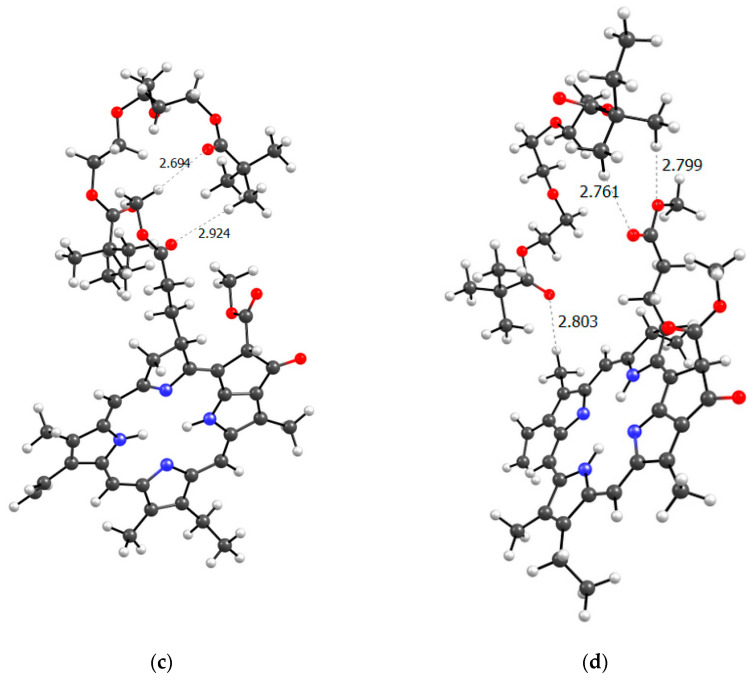
Optimized geometries of MPP dimers with TEGDM (**a**–**d**) units of VP-AA-TEGDM terpolymer.

Figure 17, Figure 18 and Figure 19 show all the possible dimers formed by the monomer units of the terpolymer and MPP in all different possible ways.

From the structure of the obtained dimers, it is clear that the shortest hydrogen bonds are formed with the AA unit (OH group hydrogen atom, MPP oxygen atoms), the shortest bond among them, according to DFT calculations, is between the hydrogen of the OH group of AA and the oxygen of the C=O ring of MPP (Figure 17). In addition to this, bonds are formed due to the C=O oxygen atom of the AA unit and the hydrogen of MPP. That is, we can say that this unit is the main to form bonds between the terpolymer and MPP. VP units can bind due to the oxygen of the lactam ring, as we demonstrated earlier [46] and numerous hydrogen atoms of the CH-groups of the MPP molecule (Figure 18). Hydrogen bonds appear as additional ones due to the oxygen atoms of MPP. As usual, the weakest bonds [47] are formed by the TEGDM unit (Figure 19), but at the same time, bonds are more often observed due to the hydrogen atoms of C—H groups.

**Figure 20:**
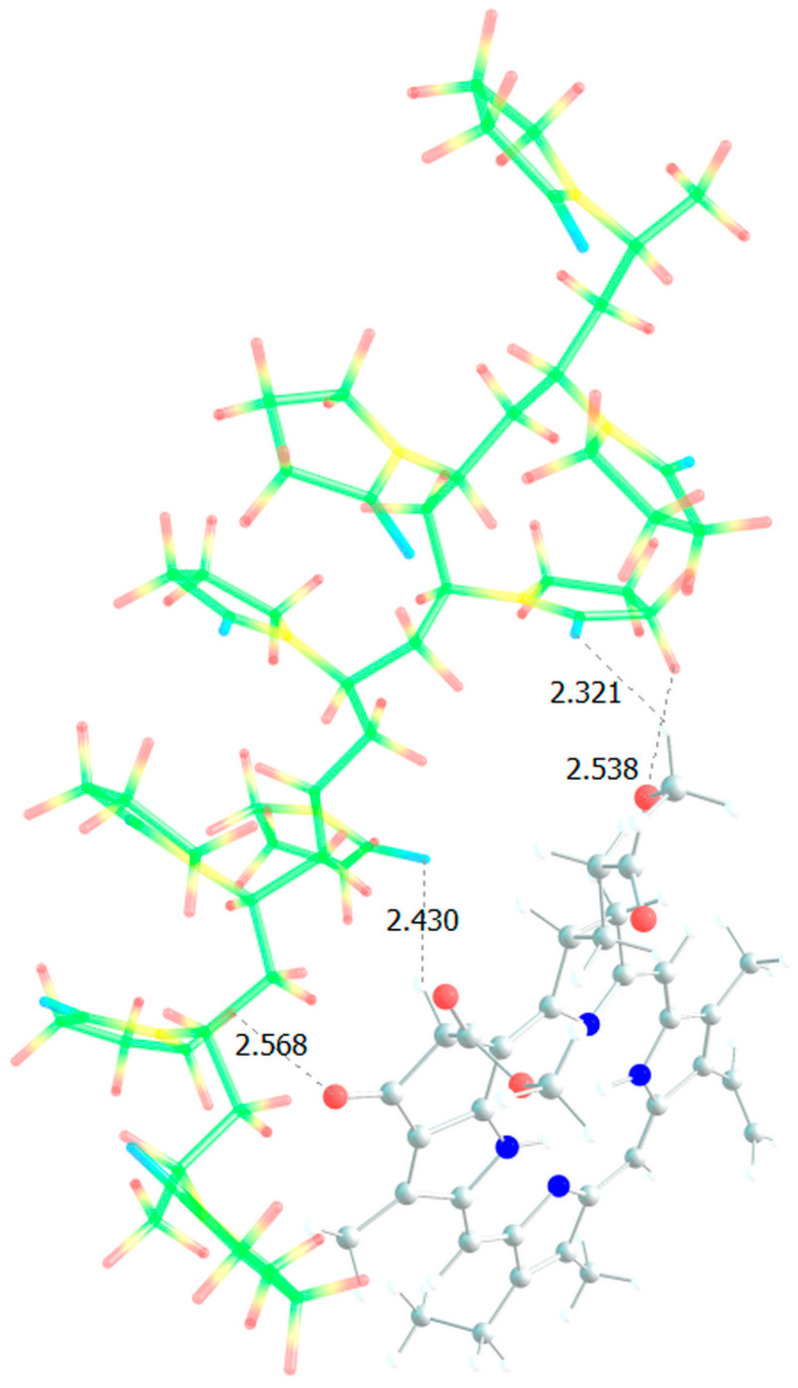
Optimized PC4 binding site geometry.

To better understand the structure of the real (poly-VP-AA-TEGDM)-MPP system, it is necessary to consider the terpolymer section based on the available experimental data. In the terpolymer, if we average the available data, 80% are VP units and approximately 10% each are AA and TEGDM units. This means that polymer chains contain sections consisting only of VP units. Clearly, these could be binding sites for MPP, since we have seen that the VP units easily form hydrogen bonds with them. Figure 20 shows a model of the PC4 binding site—optimized geometry of the MPP-10VP complex.

**Figure 21:**
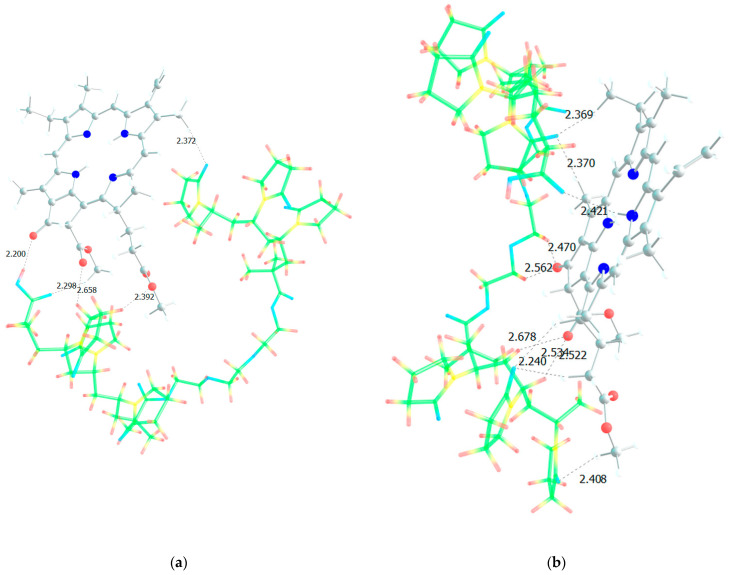
Optimized geometries of possible (poly-VP-AA-TEGDM)-MPP binding sites (**a**,**b**).

It is obvious that hydrogen bonds are formed, as we observed for dimers, stronger due to the oxygen atoms of the lactam ring, and weaker due to the oxygen atoms of the MPP. That is, the PC4 system may well exist according to calculations. However, we have a terpolymer, and it is interesting to predict how the introduction of additional units will affect it. So far, from the analysis of the dimers structure, we can believe that the introduction of AA units should strengthen the connection of MPP with the terpolymer, which cannot be said about TEGDM units. To make the comparison correct, we will again take 10 units, but replace 2 of them with AA and TEGDM units. We know that TEGDM is ~4 times more active than VP and ~50 times more active than AA. Most likely, the TEGDM unit will be either at the beginning of the chain or in the center (the most active), and the AA units will be at the end of the terpolymer chain (the most inactive). As a result, we obtain a structure containing both additional units, shown in Figure 20. Next, an attempt was made to find possible strong MPP structures with this resulting site (Figure 21).

It is easy to see that with such a binding site, the MPP molecule forms more bonds and the strongest is observed to be OH (AA)...CO(MPP), as in the previously modelled dimers. Obviously, the VP-AA-TEGDM-MPP system is stronger than the PC4 one. Quite strong, unlike model dimers, H(TEGDM)-O(MPP) bonds are added to the C=O(VP)-H(MPP) bonds. As is known, the addition of AA and TEGDM units to the composition of polymer chains will lead to the formation of a terpolymer that will have changed properties and structure compared to PVP. Copolymerization of these monomers with VP will lead to a branched copolymer structure, since acrylic acid forms side chains, and triethylene glycol dimethacrylate will contribute to the formation of soft segments in the polymer structure. Moreover, due to the addition of acrylic acid, the copolymer may have increased adsorption capacity and will be more compatible with some other compounds or surfaces due to changes in the chemical nature of the polymer. Quantum chemical calculations of the binding sites of the guest molecule MPP with (poly-VP-AA-TEGDM) chains explain this by the formation of a larger number of hydrogen bonds and their greater strength. The results of quantum chemical modeling make it possible to predict the structure of MPP nanosized systems and their use in biological experiments.

Thus, we have developed polymer compositions of a fluorescent dye, methyl pheophorbide a based on ternary copolymers of N-vinylpyrrolidone with different structures, and physicochemical properties, which, being dissolved in aqueous media, exist in the form of polymer nanoparticles loaded with ca. 1.5–3% dye. There are intermolecular bonds between the molecules of the encapsulated dye and the functional groups of the terpolymer that hold it in the polymer matrix.

### 2.5. Cytotoxicity and Intracellular Accumulation of MPP Polymer Compositions (PC1-PC7)

**Figure 22:**
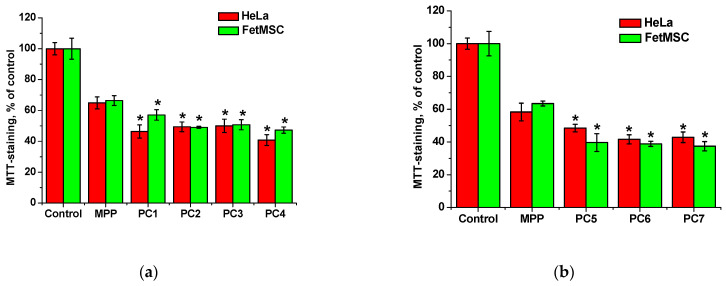
Effect of native MPP at concentrations of 15 (**a**) and 34 (**b**) mg mL^−1^, as well as polymer compositions with MPP at the concentration of 1 mg mL^−1^ on the viability of FetMSC and HeLa cells according to the results of MTT staining after 72 h of action. The significance of differences is shown relative to the control as * *p* < 0.05.

To study the penetration and accumulation of polymer particles in cells, polymer compositions of MPP were used, obtained by two methods and differing in the content of MPP. The introduction of MPP into polymer compositions can significantly change their biological activity, including cytotoxic properties. On the other hand, the structure and characteristics of terpolymers may influence the ability of MPP to accumulate in cells. The effects of native MPP and PC1-PC4, as well as PC5-PC7 on the viability of FetMSC and HeLa cells were investigated. The studied compositions at a maximum concentration of 1 mg mL^−1^ (MPP content either 15 or 34 mg mL^−1^) were compared with native MPP at similar concentrations with exposure as long as 72 h. Main results are presented in Figure 22.

From the data shown in Figure 22, it follows that MPP, regardless of concentration, causes a decrease in the viability of both non-tumor and tumor cells by ~40% relative to the control. The cytotoxic effect of MPP in polymer compositions was enhanced in all cases. Since this effect cannot be explained by the cytotoxic activity of the copolymers (Figure 6), it can be assumed that the nanoparticles significantly influenced the accumulation of MPP in cells. The effect of lower (0.03 to 0.5 mg mL^−1^) concentrations of polymer compositions with MPP on the viability of non-tumor and tumor cells was investigated (Figure S6). It was found that compositions with different MPP contents differed significantly in the severity of the cytotoxic effect. So, polymer compositions PC1, PC2, PC3 containing ~1.5% MPP at low concentrations did not cause a significant decrease in cell viability, while similar polymer compositions PC5, PC6, PC7 containing ca. 3.4% MPP even at the minimum concentration reduced MTT staining by 20–30% relative to the control. Interestingly, compositions containing 1.5% MPP were less toxic to non-tumor cells (Figure S6a) compared to tumor ones (Figure S6b). It can be assumed that this effect is associated with a more rapid accumulation of nanoparticles in tumor cells. Another interesting fact is that the use of the PC4 polymer composition based on PVP led to the same decrease in cell viability of both lines as the use of the PC5-PC7 polymer compositions with 3.4% MPP. Figure S6 shows that curves 4–6 and 7 almost completely coincide.

**Figure 23:**
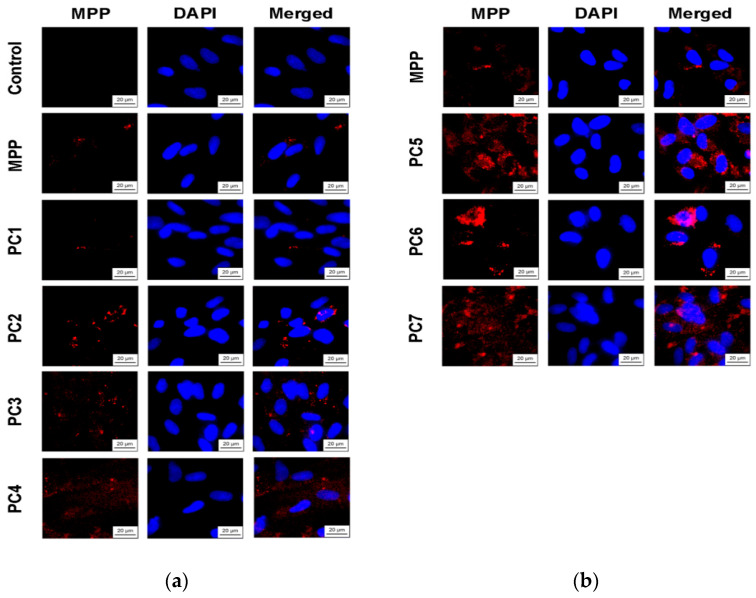
Accumulation of native MPP and PC1-PC7 in non-tumor FetMSC cells after 6 h of action: MPP concentration of 15 mg mL^−1^ (**a**) and 34 mg mL^−1^ (**b**).

**Figure 24:**
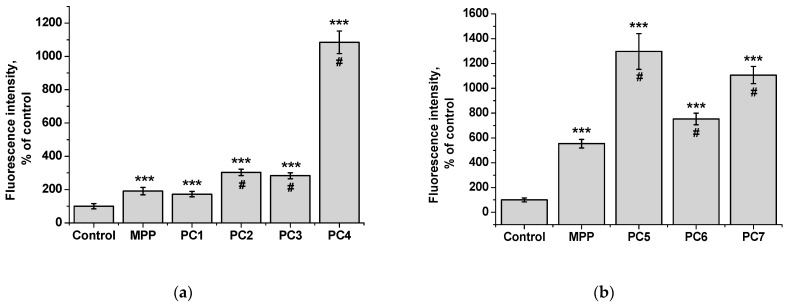
Fluorescence intensity of native MPP and MPP-polymer compositions in non-tumor FetMSC cells after 6 h of action: MPP concentration of 15 mg mL^−1^ (**a**) and 34 mg mL^−1^ (**b**) according to the results of quantitative analysis of fluorescence microscopy. The significance of differences is shown relative to the control as *** *p* < 0.001; relative to MPP as ^#^ *p* < 0.05.

**Figure 25:**
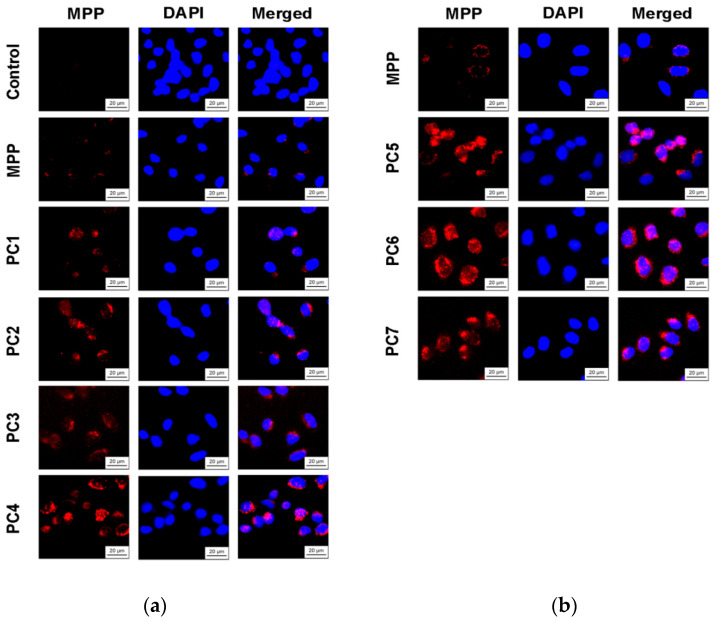
Accumulation of native MPP and PC1-PC7 in non-tumor HeLa cells after 6 h of action: MPP concentration of 15 mg mL^−1^ (**a**) and 34 mg mL^−1^ (**b**).

**Figure 26:**
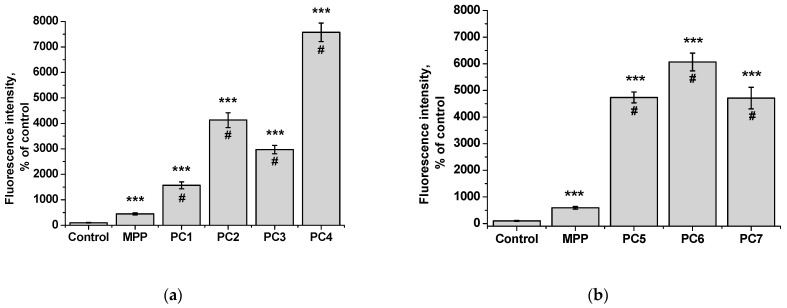
Fluorescence intensity of free MPP and PC1-PC7 in non-tumor HeLa cells after 6 h of action: MPP concentration of 15 mg mL^−1^ (**a**) and 34 mg mL^−1^ (**b**) according to the results of quantitative analysis of fluorescence microscopy. The significance of differences is shown relative to the control as *** *p* < 0.001; relative to MPP as ^#^ *p* < 0.05.

Since we assumed that the use of nanoparticles significantly changes the rate of accumulation of MPP in non-tumor and tumor cells, the accumulation of polymer compositions and native MPP was studied using fluorescence microscopy at high concentrations of nanoparticles (1 mg mL^−1^) and short exposure of 6 h. The microscopy results are presented in Figure 23 and Figure 25, quantitative analysis of fluorescent staining is shown in Figure 24 and Figure 26.

From the data presented, it follows that the use of nanoparticles in most cases increased the accumulation of MPP in both non-tumor and tumor cells. However, the ability to accumulate MPP nanoparticles with different cell types varied significantly. Thus, for PC1-PC3 in FetMSC cells, an increase in fluorescence intensity was observed by 2–3 times relative to the control (which is close to the indicators of native MPP), and in tumor cells—by 16–30 times. For the PC5-PC7, an increase of 8–13 times relative to the control was detected in non-tumor cells, while in HeLa cells an increase of 50–60 times was detected. As in the case of the effect on cell viability, PC4 nanoparticles in terms of MPP accumulation were close not to the group of polymer compositions containing 1.5% MPP, but with 3.4% MPP. Moreover, this effect was manifested in both cell lines. If one is to compare the data on the cytotoxicity of nanoparticles with data on their intracellular accumulation, one can conclude that polymer compositions containing 3.4% MPP cause a significant increase in the content of MPP in cells, which is accompanied by an increase in their toxicity for both types of cells. The use of a polymer composition containing linear PVP (PC4) as a copolymer significantly increased the intracellular accumulation of MPP compared to compositions based on terpolymers. This is likely due to the rapid release of MPP from PVP-based polymer particles and the resulting increase in fluorescence. From polymer nanoparticles based on terpolymers, densely packed macromolecular structures, the release of MPP occurs more slowly and its fluorescence is lower during the observation period. This assumption is based on data from studying the interaction of polymer nanoparticles with biological objects such as liposomes and highly diluted mouse subcellular brain homogenate [27] and the observed increase in MPP fluorescence as a result of release from NPs; the release rate depends on the structure of the polymer matrix. Thus, nanosized systems based on terpolymers have great prospects from the point of view of prolonged action of MPP. By controlling the composition of copolymers, their degree of branching and the density of molecular packaging, it is possible to effectively regulate the rate of release of MPP and the duration of its action as a fluorescent diagnostic agent and a photosensitizer in photodynamic therapy of oncological and microbial diseases.

## 3. Materials and Methods

### 3.1. The Materials

#### 3.1.1. Terpolymers of N-Vinylpyrrolidone with (Di)methacrylates and Linear PVP

We used N-vinylpyrrolidone (99%, Acros Organics), which was pre-purified by vacuum distillation. TEGDM (95%) and AA (98%, both Aldrich, St. Louis, MI, USA) were used as received. 2,2′-Azo-bis-isobutyronitrile (AIBN) purified by recrystallization from ethanol was used as an initiator. Ethanol was previously purified by distillation. The precipitant *n*-hexane (“reagent grade”) was used without purification.

The synthesis of CPL1-CPL-3 terpolymers and linear PVP was carried out in a three-neck flask equipped with a reflux condenser and a thermometer, with continuous bubbling of argon for 2 h at 80 °C in a thermostat. All components of the reaction mixture were introduced simultaneously. The reagents content in the solvent was ~20 wt %. The initiator concentration was 0.02 mol L^−1^. Terpolymerizations were homogeneous, and the terpolymers were completely soluble in ethanol. Under the same conditions, linear PVP was obtained.

Terpolymers isolation was carried out by precipitation from their solutions in ethanol with a tenfold excess of the precipitant, *n*-hexane. After its evaporation, the terpolymers were dried from the solvent to constant weight in air and in vacuo at 60 °C. The terpolymers were glassy products.

#### 3.1.2. Encapsulation of Methyl Pheophorbide a into NPs

To obtain terpolymer compositions containing a fluorescent probe, a water-insoluble dye, methyl pheophorbide a, was used. It fluoresces in the red region of the spectrum [27].

Encapsulation of MPP into polymer particles was carried out in two ways. In the first case to obtain PC1-PC3 compositions, 2.4 mL of MPP solution in toluene (0.7 mg mL^−1^) was added dropwise to 32 mL of the respective terpolymer (CPL1-CPL-3) solution (3.5 mg mL^−1^) in IPA with constant stirring using a magnetic stirrer. The terpolymer mass was 112 mg, and the MPP mass was 1.68 mg. For comparison, a composition PC4 based on PVP was prepared. IPA and toluene were evaporated in air, dried in an oven at 60 °C for 1 h, and evacuated to a constant weight.

In the second case, a diluted solution of terpolymers in IPA (1.75 mg mL^−1^) was used. A solution of MPP in DMSO (0.8 mg mL^−1^) was added dropwise to them with constant stirring using a magnetic stirrer. In both cases, visually transparent solutions were obtained; organic solvents were evaporated at a room temperature and in vacuo. The resulting films of MPP compositions based on CPL1, CPL2 and CPL3 i.e., PC5-PC7 were dried at room temperature and 60 °C and dissolved in water or PBS. Their water solutions (1 mg mL^−1^) were clear or slightly opalescent at higher PC5-PC7 concentrations.

Absorption spectra of water solutions of encapsulated dye in polymer particles were recorded using a Specord M40 spectrophotometer (Germany). The cuvette thickness was 1 cm. To determine the content of MPP in terpolymer compositions, a calibration dependence of the optical density of Q-band on MPP concentration in the range 10^−5^–10^−4^ M was plotted, and the molar extinction coefficient was determined. Solutions of polymer compositions were prepared in DMSO. From the obtained data and the calculated content of MPP in the composition; the efficiency of MPP encapsulation was calculated as the relation of encapsulated mass of MPP by total mass of the dye in the PC synthesis.

### 3.2. The Methods

#### 3.2.1. Elemental Analysis

Using elemental analysis, the *N* content in the resulting VP copolymers was determined using a Vario MICRO cube (Elementar Analysensysteme GmbH, Langensel-bold, Germany, 2007).

#### 3.2.2. IR- and ^1^H NMR Spectroscopy

IR spectra of terpolymers were recorded in the attenuated total internal reflection mode on a Bruker α FTIR device (Germany) in the range of 400–4000 cm^−1^, the number of scans was 16, the cuvette length was 1 cm.

^1^H NMR spectra of terpolymer solutions in deuterated chloroform (6 mg mL^−1^) were recorded on a superconducting pulsed broadband two-channel NMR spectrometer AVANCE III 500 MHz Bruker Biopin (Germany).

#### 3.2.3. Size-Exclusion Chromatography

The absolute molecular weights of the terpolymers VP-AA-TEGDM were determined by size exclusion chromatography (SEC) using a Waters liquid chromatograph (2 columns PS-gel, 5 μm, MIXED-C, 300 × 7.5 mm) equipped with a refractometric detector and a WYATT DAWN HELEOS II multi-angle light scattering detector, Wyatt λ = 658 nm (Waters, Milford, MA, USA). The eluent was *N*-methylpyrrolidone with the addition of lithium chloride (1 wt%), which prevents the aggregation of macromolecules in a polar solvent. The measurement temperature was 70 °C, the elution rate was 1 mL min^−1^. The d*n*/d*c* values were determined from the data of the multi-angle light scattering detector. All polymer solutions were pre-filtered through filters with a pore diameter of 0.2 µm. The absolute average molecular weight of the terpolymer was obtained from light scattering detector data using Astra software, version 5.3.2.20.

#### 3.2.4. Dynamic Light Scattering

The behavior of the terpolymers in aqueous solutions was studied by the dynamic light scattering (DLS) method. Before measurements, the vials were washed with a solvent purified through a 0.45 µm PES filter and thermostated at a given temperature for 20 min. The sizes of NPs-MPP in phosphate-buffered saline (PBS) at 25 °C were determined at different concentrations. The measurements were carried out using a Photocor Compact setup (Photocor Instruments Inc., Beltsville, MD, USA), equipped with a diode laser (λ 654 nm), at a detection angle of 90°. Experimental data were processed using DynaLS software, version 2.8.3. The hydrodynamic radii, R_h_, of the terpolymers were calculated using the Einstein-Stokes equation. From the dependence of the intensity of scattered light and the hydrodynamic radius of scattering centers on temperature, conclusions were drawn about the thermal sensitivity of the copolymers.

#### 3.2.5. TEM Study of PC1 and PC3 from Water Solution

TEM images of the PC1 and PC3 were obtained from water solutions using the JEM-2100 microscope (JEOL, Tokyo, Japan) operating at 200 kV. The sample concentration was 1 mg mL^−1^. A drop of solution was applied to a copper grid covered with a carbon film and dried in air at room temperature. Under these conditions, water strongly bound to the polymers, apparently, was retained in the samples.

#### 3.2.6. Quantum Chemical Calculations

All quantum chemical calculations were carried out in the Gaussian 09 program [48] using several methods depending on the complexity of the object. Density functional theory (DFT) with full geometry optimization was used to calculate the original MPP guest molecule and its dimers. The TPSSh hybrid functional and the 6-31G* basis set were used as a method and basis. To optimize the geometry of binding sites, the semi-empirical AM1 method was used. There are no imaginary vibration frequencies in the calculation results; all optimized structures correspond to the minimum potential energy.

#### 3.2.7. The Study of the Cytotoxicity of Copolymers, Polymer Compositions and MPP on Normal and Tumor Cells

The cytotoxicity was studied on non-tumor cells FetMSC (human mesenchymal stem cells), Vero (renal epithelium of the African green monkey), and tumor cells HeLa (human cervical adenocarcinoma, clone M-HeLa), obtained from the collection of the Institute of Cytology of the Russian Academy of Sciences.

Cell cultivation was carried out according to standard methods in an atmosphere of 5% CO_2_ and a temperature of 37 °C in DMEM (Vero), EMEM (HeLa) or DMEM/F-12 (FetMSC) medium with the addition of 10% fetal calf serum (BioWest), 50 U mL^−1^ penicillin and 50 mg mL^−1^ streptomycin.

The study of the cytotoxic properties of copolymers, polymer compositions and MPP was carried out using the MTT test. Cells were seeded into 96-well culture plates at a concentration of 5’ 10^4^ cells∙mL^−1^ (HeLa and Vero), 10^5^ cells∙mL^−1^ (FetMSC). The compounds were added to the culture medium 24 h after sieving at a maximum concentration of 1 mg∙mL^−1^ for terpolymers and polymer compositions, and 1.5 or 3.4 mg∙mL^−1^ for MPP. After 72 h of incubation, MTT dye (3-(4.5-dimethylthiazol-2-yl)-2.5-diphenyl-2H-tetrazolium bromide) was added to the culture medium at a concentration of 0.5 mg∙mL^−1^. The resulting formazan crystals were dissolved in 100% DMSO. Optical density measurements were carried out at a main wavelength of 570 nm and a background wavelength of 620 nm using a Spark 10M multifunctional tablet reader (Tecan, Männedorf, Switzerland).

To study the intracellular accumulation of polymer compositions and MPP, cells were seeded at a concentration of 2 × 10^5^ mL^−1^ onto coverslips in 6-well plates in 2 mL the standard incubation medium. After 24 h, MPP or its polymer compositions were added to the culture medium at concentrations of 1.5 or 3.4 mg mL^−1^ (calculated on MPP). After 6 h, the culture medium was removed, the cells were washed three times with PBS (pH 7.4) and fixed with a 4% solution of paraformaldehyde (Acros Organics) in PBS for 30 min. Cell nuclei were stained with the fluorescent dye DAPI (Serva) in PBS for 15 min. Samples were visualized using an AxioScope A1 microscope (Zeiss) and an A-Plan 40×/0.65 objective using fluorescent filter sets Fs 49 DAPI (EX G 365, EM BP 445/50), Fs 45 HQ TexasRed (EX BP 560/40, EM BP 630/75). Fluorescence intensity was analyzed in 5 fields of view containing 100 cells. Images of the samples were obtained using an AxioCam MRc5 camera and processed in the ZEN 2 lite program; quantitative fluorescence measurements were performed in the ImageJ program (NIH).

Statistical processing of the data from biological experiments was carried out using the GraphPad Prism 5 and Anova Calculator programs. Data from three independent experiments are presented as mean (M) ± standard deviation (SD). The significance of differences between groups was assessed using analysis of variance (ANOVA). Values at *p* < 0.05 were considered as statistically significant.

## 4. Conclusions

In this work, new terpolymers of N-vinylpyrrolidone with acrylic acid and triethylene glycol dimethacrylate of various molar compositions, absolute molecular weight from 30 to 500 kDa and a hydrodynamic radius of less than 100 nm, low toxicity to non-tumor (Vero, FetMSC) and tumor (HeLa) cells were synthesized by radical copolymerization in ethanol. The VP-AA-TEGDM terpolymer, produced at 98:2:5 molar ratio of comonomers and enriched with dimethacrylate is characterized by a high degree of branching and reacts reversibly in water to an increase in temperature above 40 °C. Based on them, polymer compositions of a hydrophobic fluorescent dye, methyl pheophorbide a, were developed and characterized. When dissolved in aqueous media, they exist in the form of polymer nanoparticles loaded with ca. 1.5–3% dye. There are intermolecular bonds between the molecules of the encapsulated dye and the functional groups of the terpolymer that hold it in the polymer matrix. This made it possible to study their penetration and accumulation in non-tumor and tumor cells using fluorescence microscopy. It was concluded that the obtained terpolymers can be used in biomedical applications for the delivery of biologically active compounds. Encapsulated MPP in terpolymers can be considered as promising photosensitizers for photodynamic therapy and fluorescent diagnostics.

## Figures and Tables

**Table 1:** Physico-chemical characteristics of CPL1-CPL3.

Terpolymer	Monomer Mixture Composition VP-AA-TEGDM, mol %	N, %	Molar Composition of Terpolymers [VP]/[AA + TEGDM], %	M_w_ (RI + MALLS), kDa	PD	R_h_, nm		
						in Water	in PBS	
CPL1	(98:2):2	9.51 ± 0.01	83.2/16.8	78.7	1.7	77	90	
CPL2	(95:5):2	9.55 ± 0.01	79.0/21.0	36.1	1.9	90	105	
CPL3	(98:2):5	8.82 ± 0.03	82.5/17.5	570.0	5.9	57	77	

## Data Availability

Data are contained within the article.

## Supplementary Materials

The following supporting information can be downloaded at: https://www.mdpi.com/article/10.3390/ijms25158422/s1.
