# Supplementary material for: New Amphiphilic Terpolymers of N-Vinylpyrrolidone with Acrylic Acid and Triethylene Glycol Dimethacrylate as Promising Drug Delivery: Design, Synthesis and Biological Properties In Vitro
Source: Int J Mol Sci. 2024 Aug 1;25(15):8422. doi: 10.3390/ijms25158422 (PMC11312434; doi:10.3390/ijms25158422)
Supplement: Supplementary file 1 [file ijms-25-08422-s001.zip › ijms-3061580-supplementary.pdf]

International Journal of *Molecular Sciences*

MDPI

Supplementary Material

Article

New Amphiphilic Terpolymers of *N*-Vinylpyrrolidone with Acrylic Acid and Triethylene Glycol Dimethacrylate as Promising Drug Delivery: Design, Synthesis and Biological Properties *in vitro*

Svetlana V. Kurmaz [1,*], Roman I. Komendant [1], Evgenia O. Perepelitsina [1], Vladimir A. Kurmaz [1], Igor I. Khodos [2], Nina S. Emelyanova [1], Natalia V. Filatova [1], Vera I. Amozova [1], Anastasia A. Balakina [1] and Alexey A. Terentyev [1]

[1] Federal Research Center of Problems of Chemical Physics and Medicinal Chemistry, Russian Academy of Sciences, 142432 Chernogolovka, Russia; jane@icp.ac.ru (E.O.P.); komendant@icp.ac.ru (R.I.K.); kurmaz@icp.ac.ru (V.A.K.); n_emel@mail.ru (N.S.E.); natasha55555@yandex.ru (N.V.F.); amozovavi@gmail.com (V.I.A.); stasya.balakina@gmail.com (A.A.B.); alexei@icp.ac.ru (A.A.T.)

[2] Institute of Microelectronics Technology and High-Purity Materials, Russian Academy of Sciences; khodos.igor@mail.ru (I.I.K.)

* skurmaz@icp.ac.ru; Tel.: +7-496-522-10-89

Citation: To be added by editorial staff during production.

Academic Editor: Firstname Lastname

Received: date
Revised: date
Accepted: date
Published: date

Copyright: © 2024 by the authors. Submitted for possible open access publication under the terms and conditions of the Creative Commons Attribution (CC BY) license (https://creativecommons.org/licenses/by/4.0/).

Figure S1. Chromatograms of CPL1−CPL3 obtained from a refractometer (**a**) and light scattering detectors data (**b**).
Figure S2. Absorption spectra of MPP (insert) in toluene (a) and DMSO (b).
Figure S3. Changes in the absorption spectra of aqueous PC solutions: PC1 (**a**), PC2 (**b**), PC3 (**c**), and PC4 (**d**) over time. Cuvette was 1 cm. Arrows indicate changes in the optical density of the Q-band.
Figure S4. The absorption spectra of PC1-PC4 in DMSO and their change over time. Their concentrations were: 1.0, 1.1, 0.96 and 0.86 mg mL^{-1}, respectively. Cuvette was 1 cm. Solid lines – freshly prepared solutions; dashed lines – after 3 months.
Table S1. The size of scattering centers of PC1-PC4 in PBS at 25 °C
Table S2. The sizes of scattering centers of PC5-PC7 in PBS at 25 °C
Figure S5. IR spectra of MPP, CPL1, PC5 (**a**) and CPL3, PC7 (**b**) powders. The IR spectrum of DMSO is shown by an arrow.
Figure S6. Effect of MPP polymer compositions at concentrations from 0.03 to 0.5 mg mL^{-1} on the viability of *FetMSC* (**a**) and *HeLa* (**b**) cells according to the results of MTT staining after 72 h of action: PC1 (curves *1*), PC2 (curves *2*), PC3 (curves *3*), PC4 (curves *4*), PC5 (curves *5*), PC6 (curves *6*), PC7 (curves *7*).

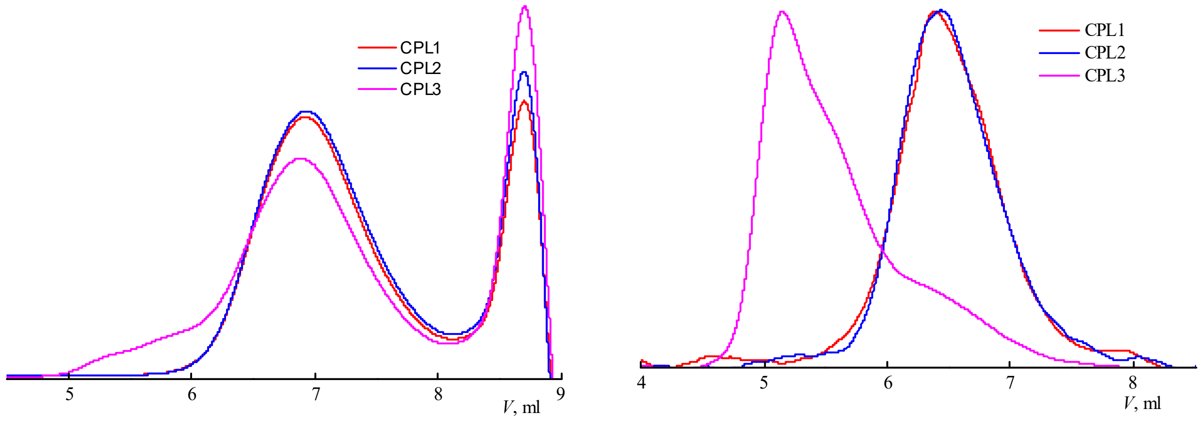

(**a**) (**b**)

Figure S1. Chromatograms of CPL1–CPL3 obtained from a refractometer (**a**) and light scattering detectors data (**b**).

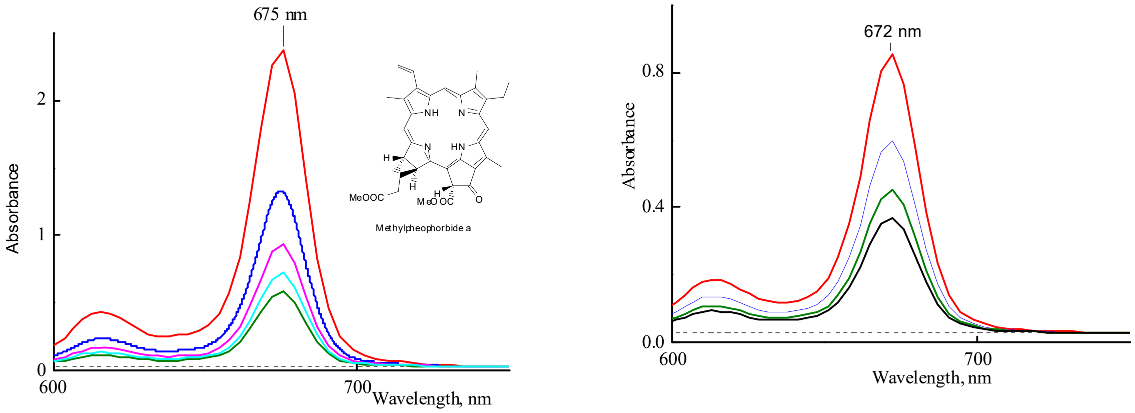

(**a**) (**b**)

Figure S2. Absorption spectra of MPP (insert) in toluene (a) and DMSO (b).

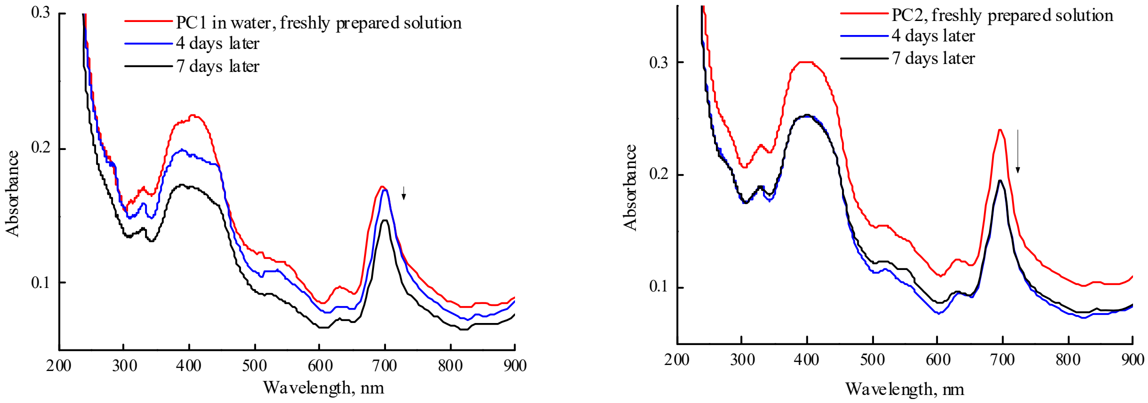

(**a**) (**b**)

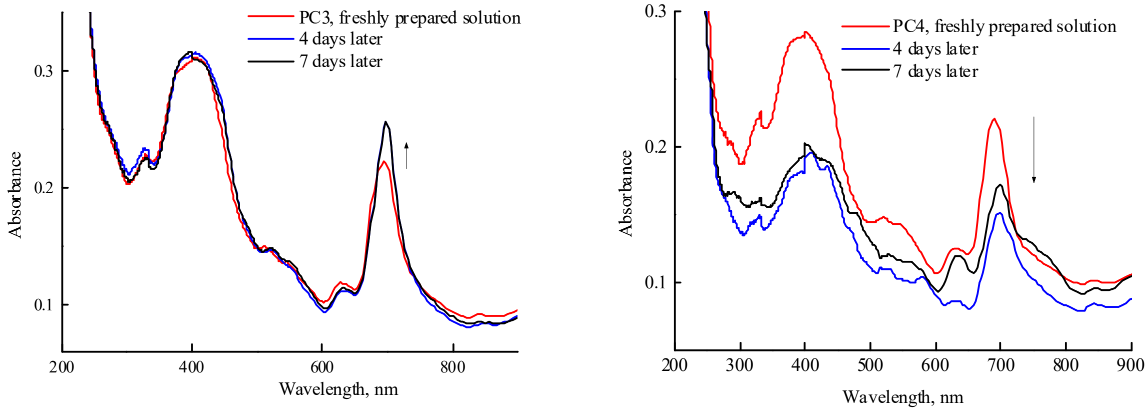

(**c**) (**d**)

Figure S3. Changes in the absorption spectra of aqueous PC solutions: PC1 (**a**), PC2 (**b**), PC3 (**c**), and PC4 (**d**) over time. Cuvette was 1 cm. Arrows indicate changes in the optical density of the Q-band.

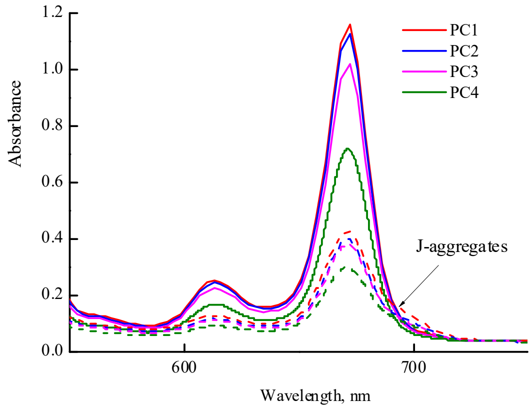

Figure S4. The absorption spectra of PC1-PC4 in DMSO and their change over time. Their concentrations were: 1.0, 1.1, 0.96 and 0.86 mg mL^{-1}, respectively. Cuvette was 1 cm. Solid lines – freshly prepared solutions; dashed lines – after 3 months.

Table S1. The size of scattering centers of PC1-PC4 in PBS at 25 °C

[PC], mg/mL	R_h, nm			
	PC1	PC2	PC3	PC4
1.0	258.9	407.9	259.2	301.6
0.5	222.8	259.1	222.9	222.9
0.25/0.25*	77.3/77.3	191.5/77.3	141.6/121.7	-

*after filtration solution through a filter (0.45 microns).

Table S2. The sizes of scattering centers of PC5-PC7 in PBS at 25 °C

[PC], mg/mL	R_h, nm		
	PC5	PC6	PC7
0.25	191.7	191.7	164.6
0.0625/0.0625*	191.7/77.4	164.6/121.6	164.6/89.9

*after filtration solution through a filter (0.45 microns).

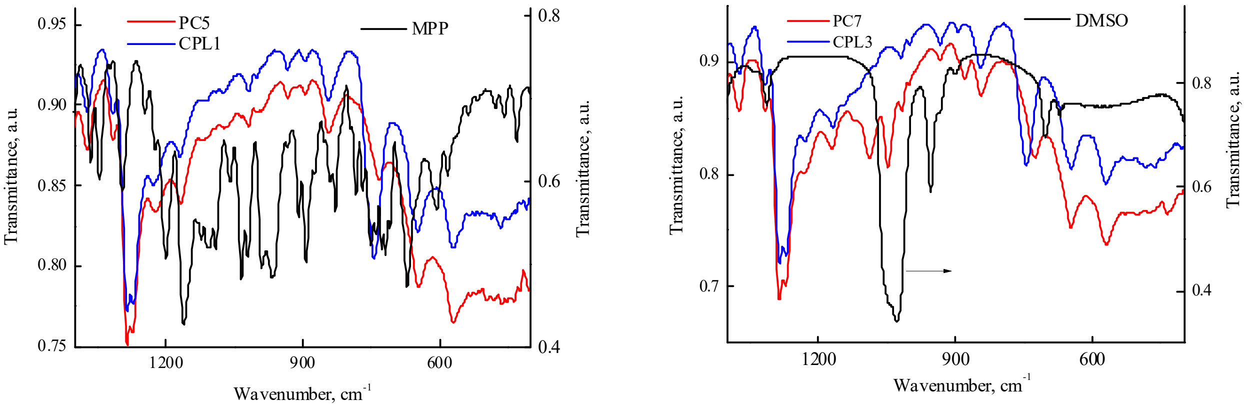

(**a**) (**b**)

Figure S5. IR spectra of MPP, CPL1, PC5 (**a**) and CPL3, PC7 (**b**) powders. The IR spectrum of DMSO is shown by an arrow.

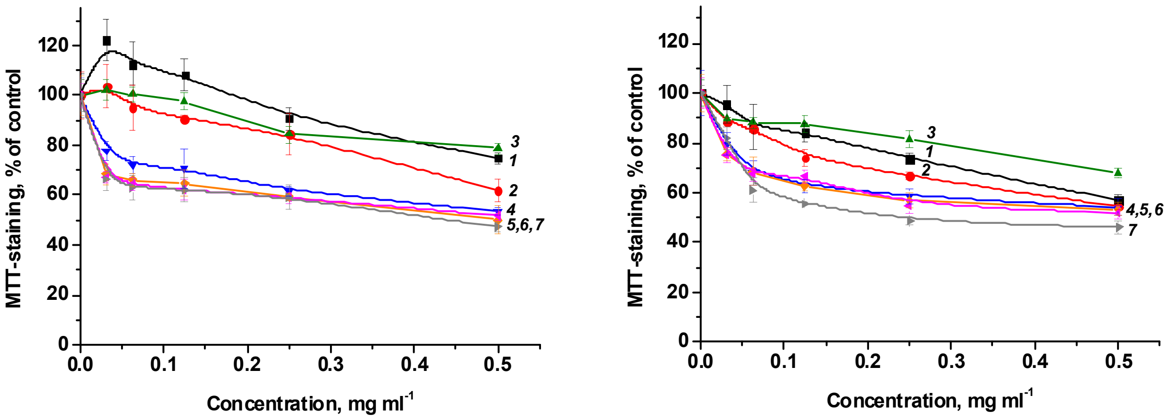

(**a**) (**b**)

Figure S6. Effect of MPP polymer compositions at concentrations from 0.03 to 0.5 mg mL^{-1} on the viability of *FetMSC* (**a**) and *HeLa* (**b**) cells according to the results of MTT staining after 72 h of action: PC1 (curves *1*), PC2 (curves 2), PC3 (curves *3*), PC4 (curves *4*), PC5 (curves *5*), PC6 (curves *6*), PC7 (curves *7*).
